# Supplementary material for: Genomic characterization of malignant progression in neoplastic pancreatic cysts
Source: Nat Commun. 2020 Aug 14;11:4085. doi: 10.1038/s41467-020-17917-8 (PMC7428044; doi:10.1038/s41467-020-17917-8)

Genomic characterization of malignant progression in neoplastic pancreatic cysts

Noë et al.

Supplementary Figure 1. Somatic mutations, phylogeny, and laser capture microdissection in MTP1. 1a. Mutations (rows) identified in the different samples (columns) in MTP1. Sample and mutation characteristics are described by the legend. The type of sequencing analysis (targeted or whole exome sequencing) performed for each sample is indicated in a track on the bottom. 1b. The inferred tumor phylogeny. The pathological characteristics for the clones are indicated by the color of the line and driver mutations are indicated at branch points. 1c. Comparison of variant allele frequencies (VAFs) in sample pairs. Each plot shows the VAFs of the corresponding samples indicated on the horizontal and vertical axes. Sample and mutation types are indicated by the colors in the legend. High VAFs of mutations shared in IPMN/MCN and cancer samples demonstrate clonal relatedness and exclude the possibility of contamination by minute amounts of cells.

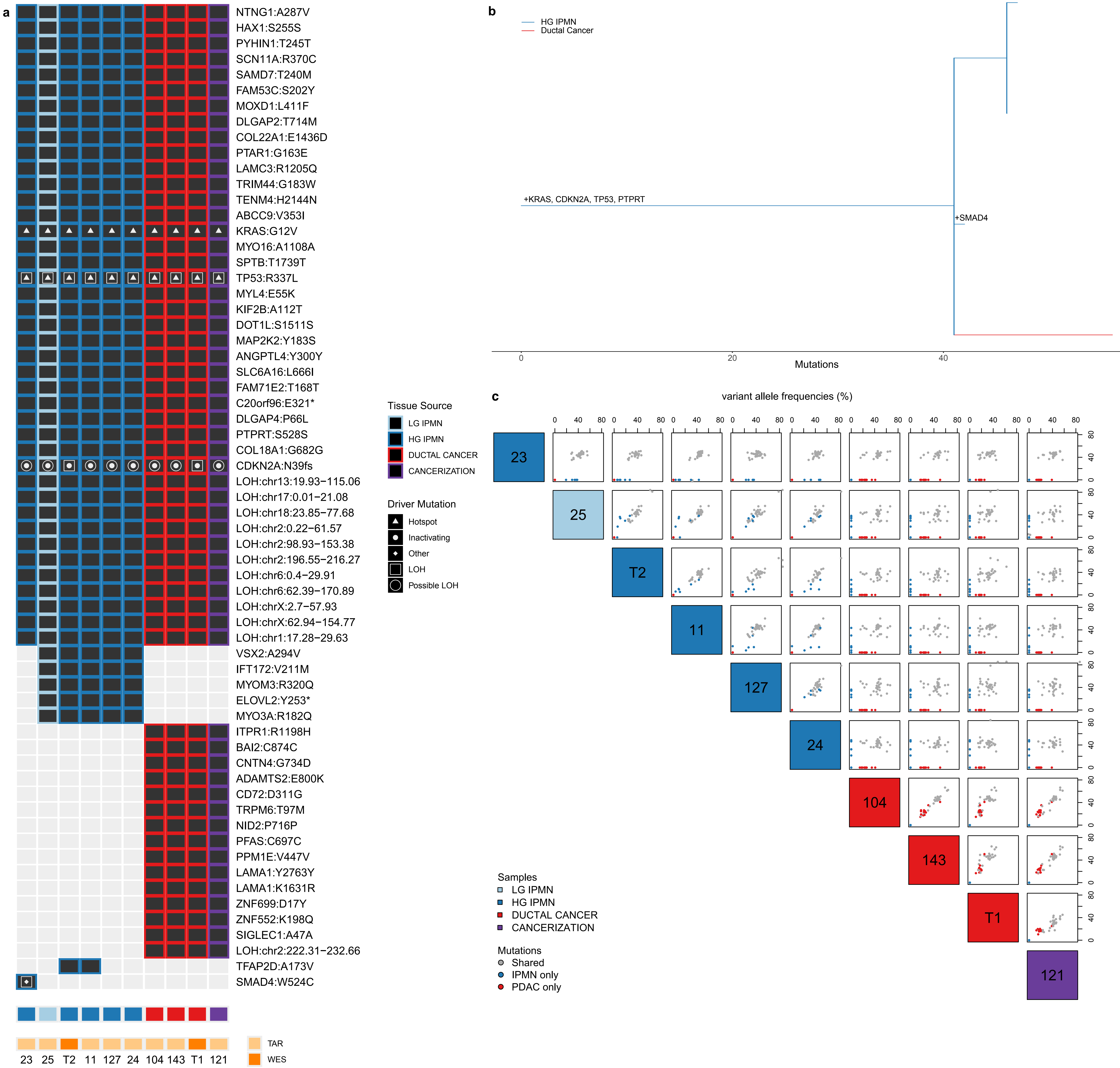

Supplementary Figure 1. Somatic mutations, phylogeny, and laser capture microdissection in MTP1. 1d. Representative images of neoplastic tissue stained by hematoxylin and eosin (H&E), as well as isolated regions before and after laser capture microdissection are shown.

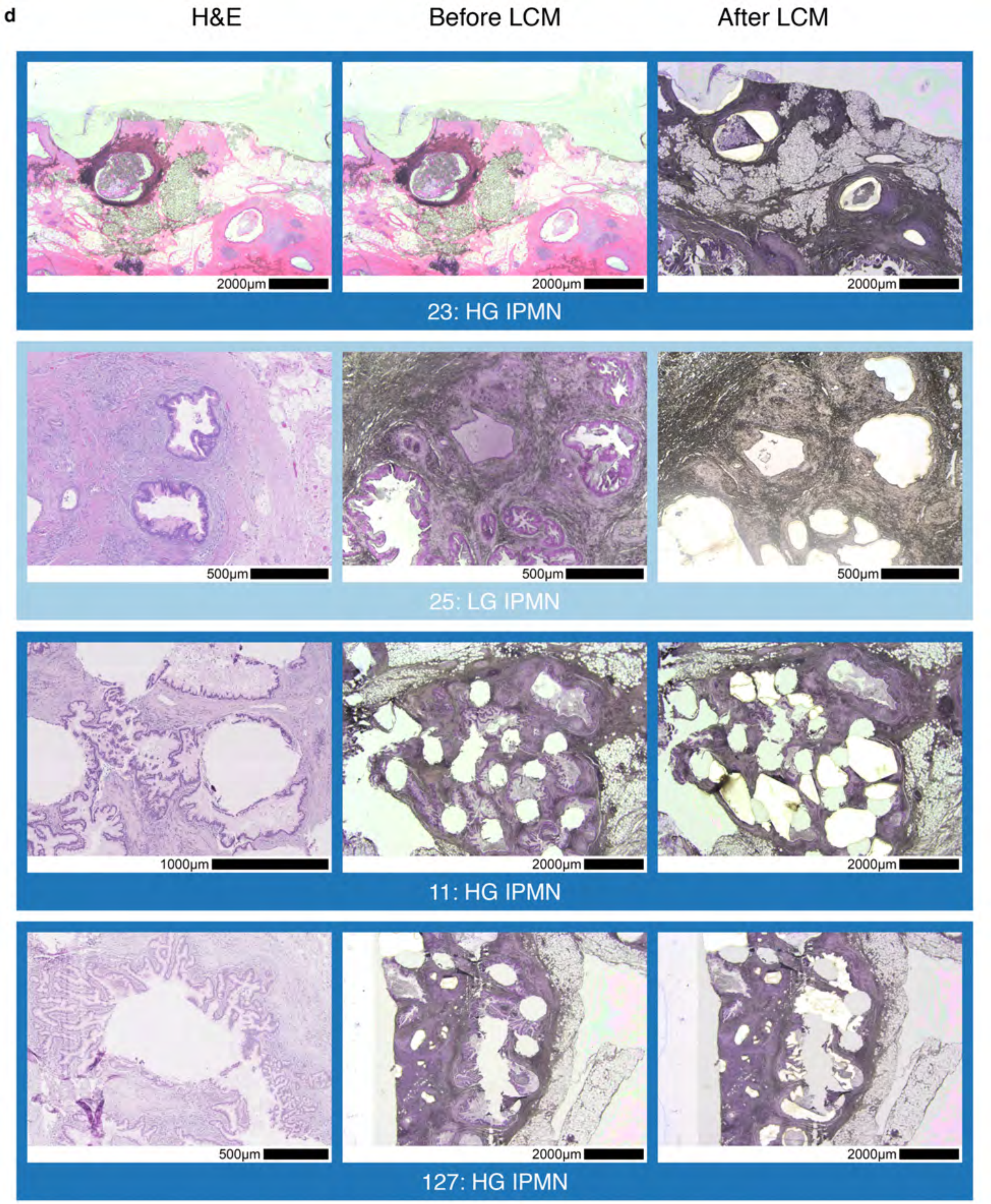

Supplementary Figure 1. Somatic mutations, phylogeny, and laser capture microdissection in MTP1. 1d. Representative images of neoplastic tissue stained by hematoxylin and eosin (H&E), as well as isolated regions before and after laser capture microdissection are shown.

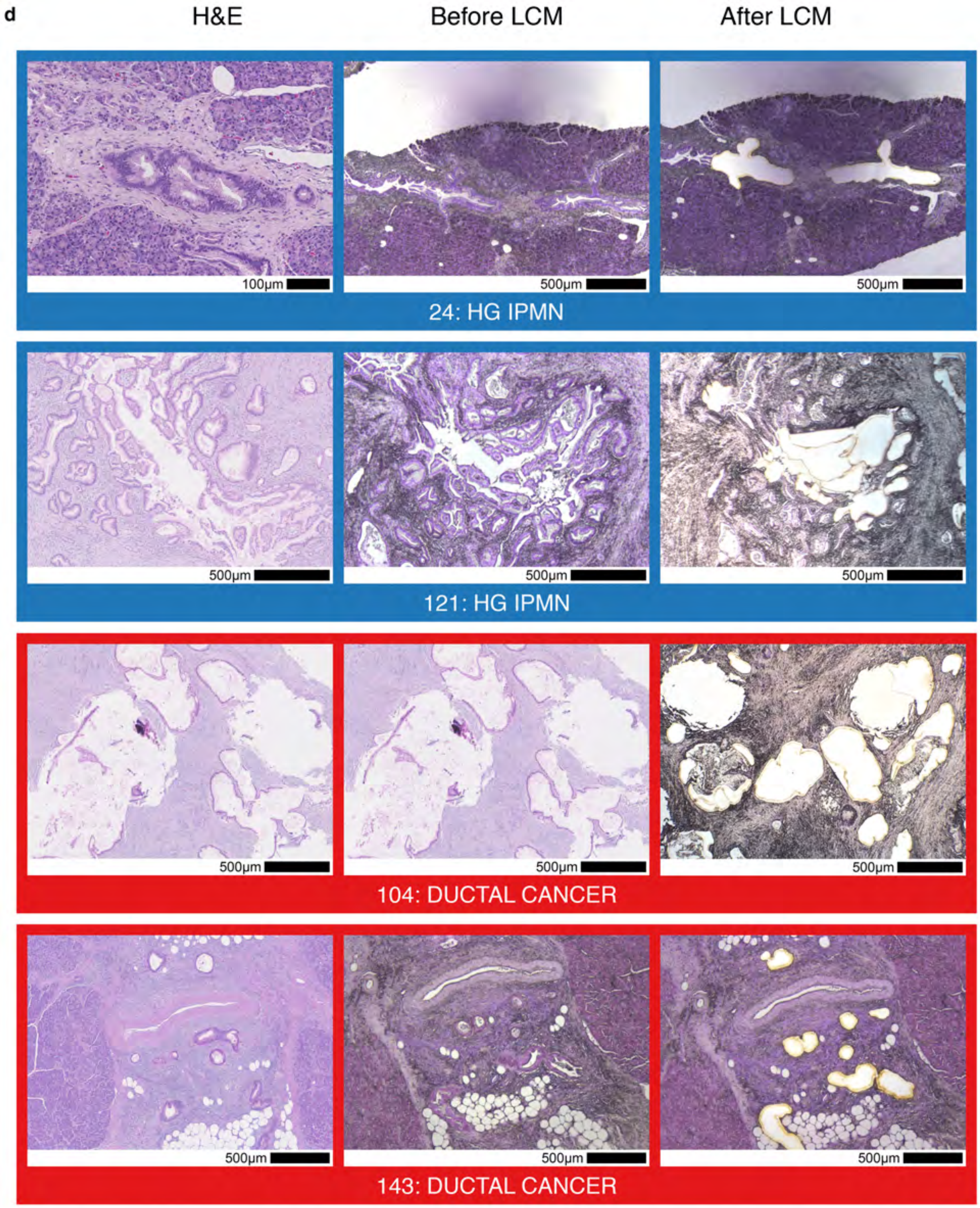

Supplementary Figure 2. Somatic mutations, phylogeny and laser capture microdissection in MTP2. 2a. Mutations (rows) identified in the different samples (columns) in MTP2. Sample and mutation characteristics are described by the legend. The type of sequencing analysis (targeted or whole exome sequencing) performed for each sample is indicated in a track on the bottom. 2b. The inferred tumor phylogeny. The pathological characteristics for the clones are indicated by the color of the line and driver mutations are indicated at branch points. 2c. Comparison of variant allele frequencies (VAFs) in sample pairs. Each plot shows the VAFs of the corresponding samples indicated on the horizontal and vertical axes. Sample and mutation types are indicated by the colors in the legend. High VAFs of mutations shared in IPMN/MCN and cancer samples demonstrate clonal relatedness and exclude the possibility of contamination by minute amounts of cells.

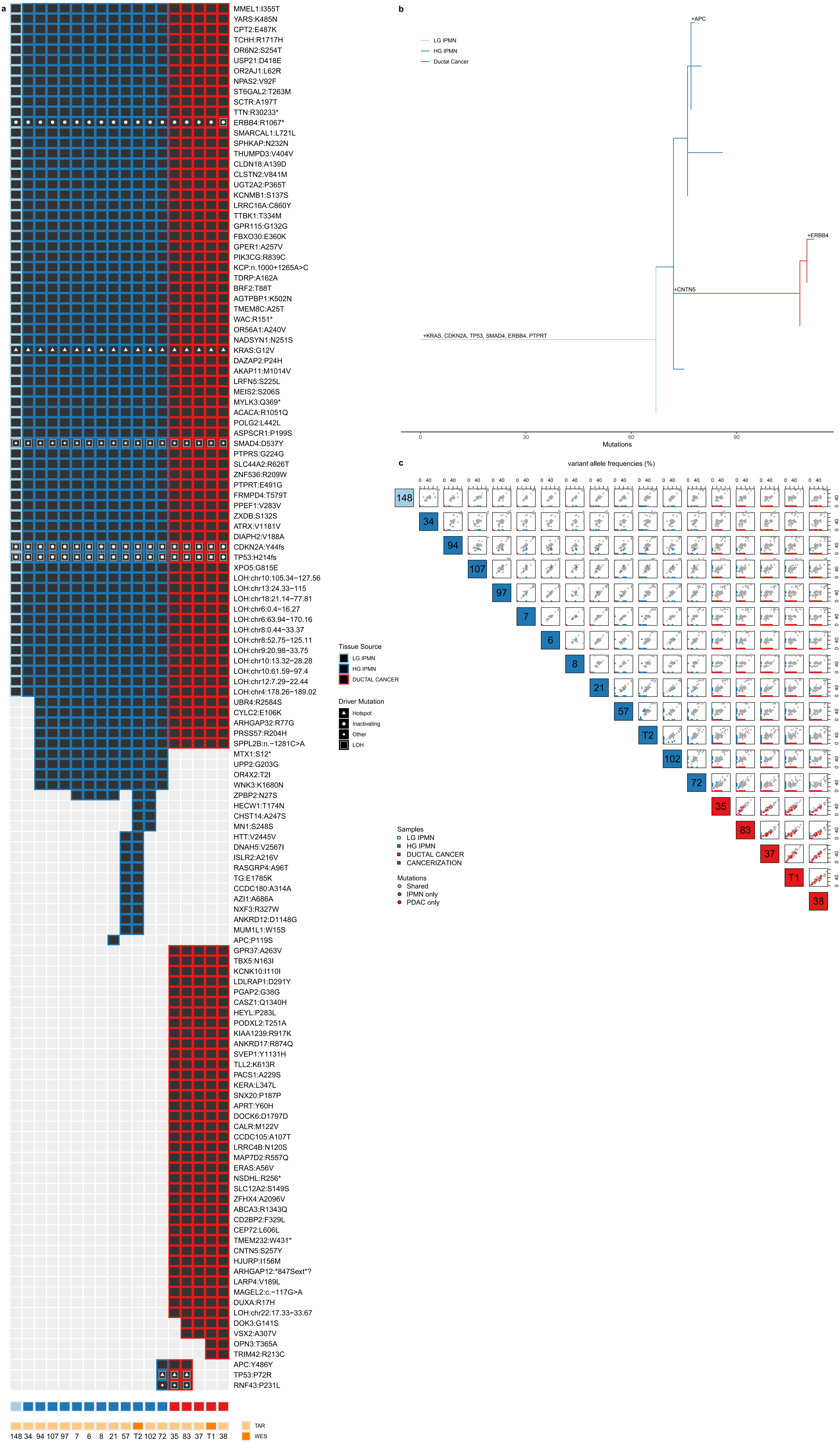

Supplementary Figure 2. Somatic mutations, phylogeny and laser capture microdissection in MTP2. 2d. Representative images of neoplastic tissue stained by hematoxylin and eosin (H&E), as well as isolated regions before and after laser capture microdissection are shown.

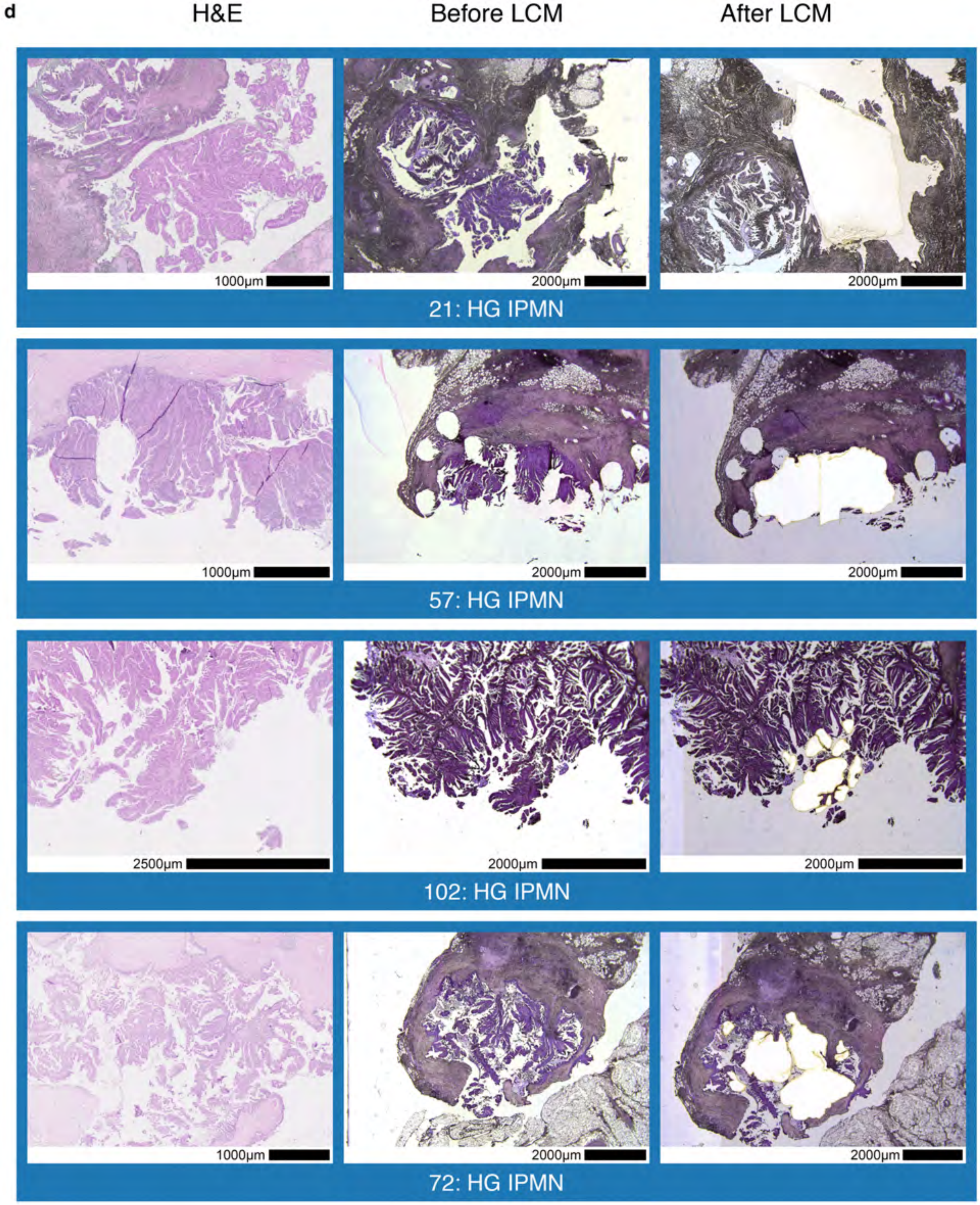

Supplementary Figure 2. Somatic mutations, phylogeny and laser capture microdissection in MTP2. 2d. Representative images of neoplastic tissue stained by hematoxylin and eosin (H&E), as well as isolated regions before and after laser capture microdissection are shown.

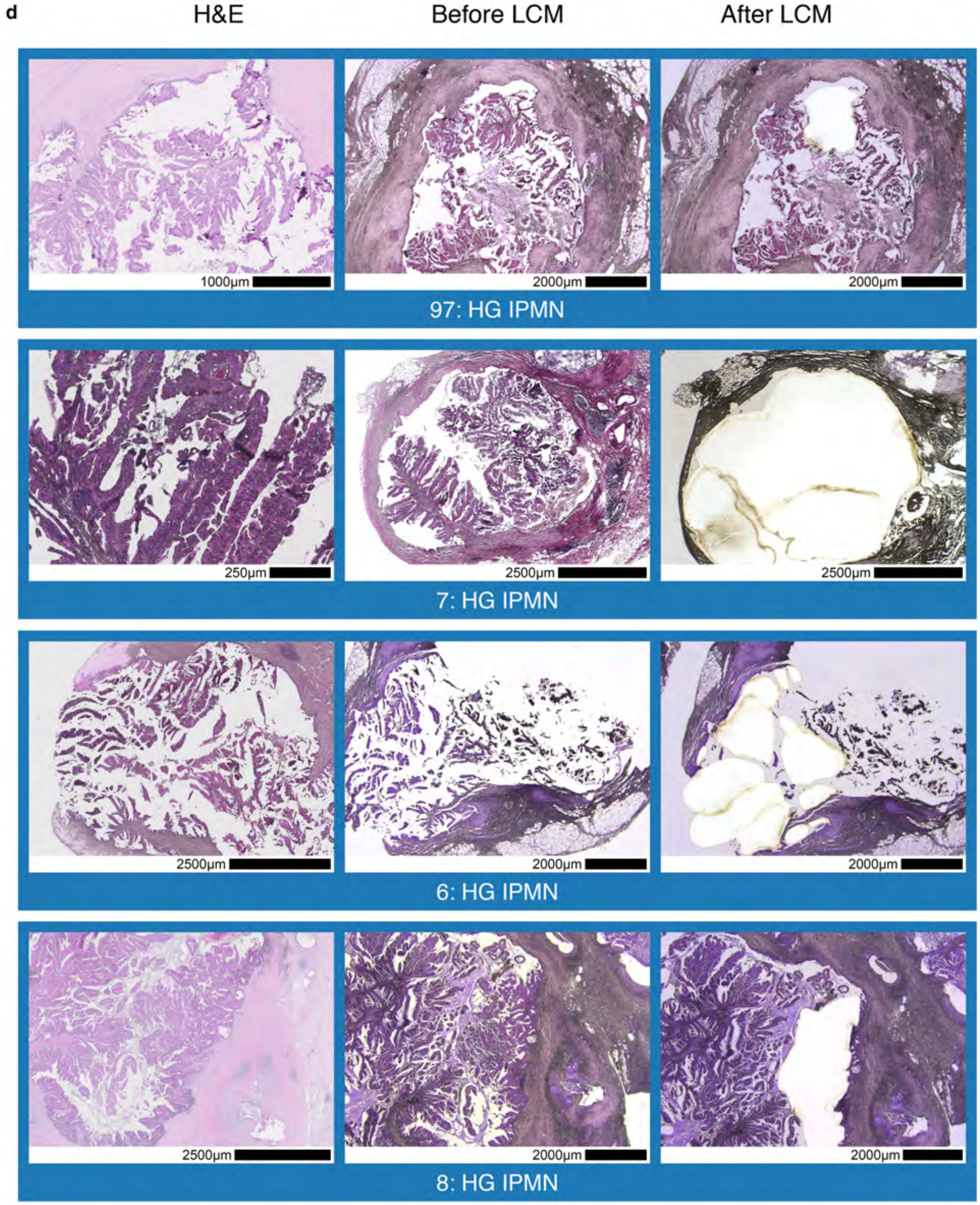

Supplementary Figure 2. Somatic mutations, phylogeny and laser capture microdissection in MTP2. 2d. Representative images of neoplastic tissue stained by hematoxylin and eosin (H&E), as well as isolated regions before and after laser capture microdissection are shown.

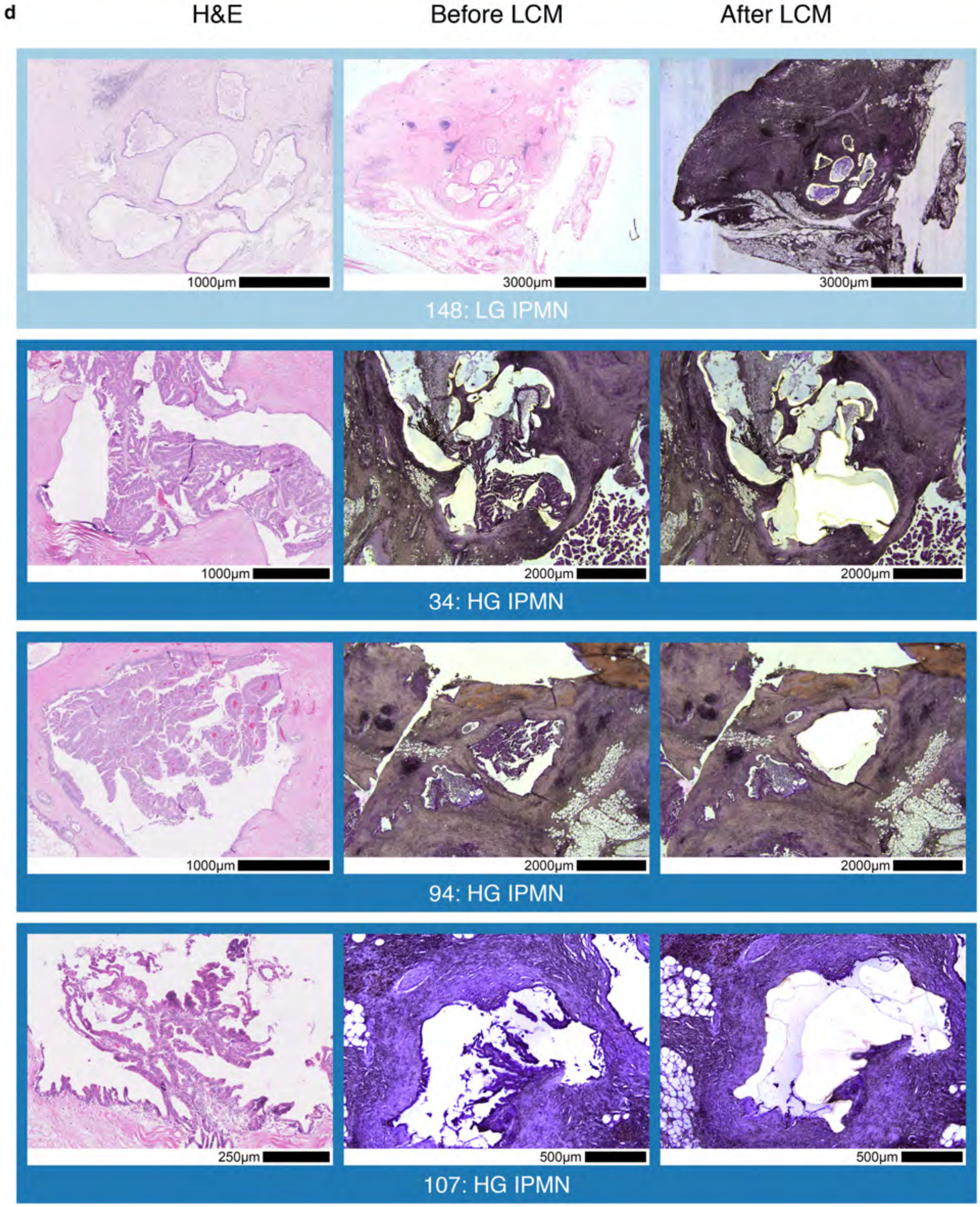

Supplementary Figure 2. Somatic mutations, phylogeny and laser capture microdissection in MTP2. 2d. Representative images of neoplastic tissue stained by hematoxylin and eosin (H&E), as well as isolated regions before and after laser capture microdissection are shown.

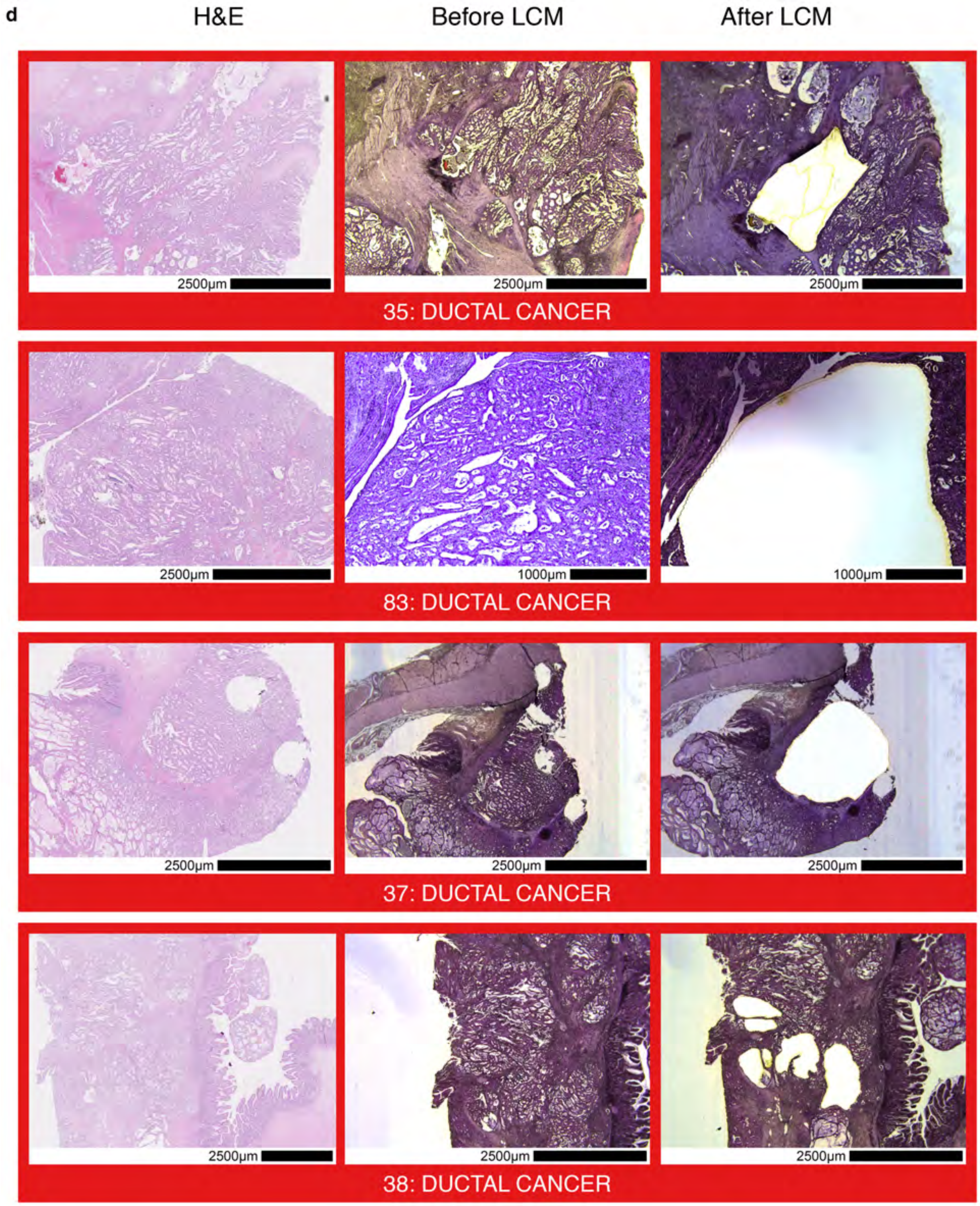

Supplementary Figure 3. Somatic mutations, phylogeny and laser capture microdissection in MTP3. 3a. Mutations (rows) identified in the different samples (columns) in MTP3. Sample and mutation characteristics are described by the legend. The type of sequencing analysis (targeted or whole exome sequencing) performed for each sample is indicated in a track on the bottom. 3b. The inferred tumor phylogeny. The pathological characteristics for the clones are indicated by the color of the line and driver mutations are indicated at branch points. 3c. Comparison of variant allele frequencies (VAFs) in sample pairs. Each plot shows the VAFs of the corresponding samples indicated on the horizontal and vertical axes. Sample and mutation types are indicated by the colors in the legend. High VAFs of mutations shared in IPMN/MCN and cancer samples demonstrate clonal relatedness and exclude the possibility of contamination by minute amounts of cells.

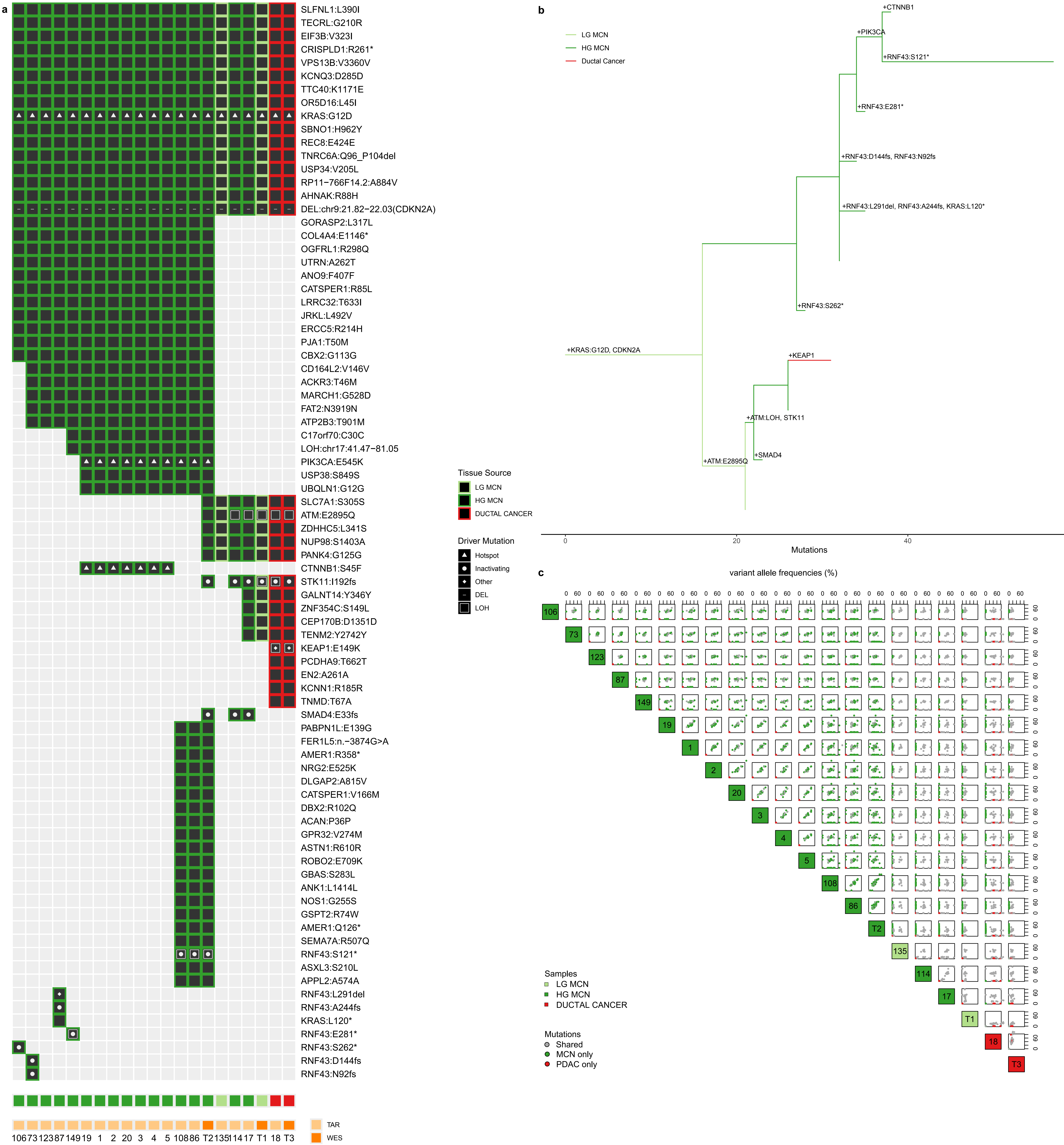

Supplementary Figure 3. Somatic mutations, phylogeny and laser capture microdissection in MTP3. 3d. Representative images of neoplastic tissue stained by hematoxylin and eosin (H&E), as well as isolated regions before and after laser capture microdissection are shown.

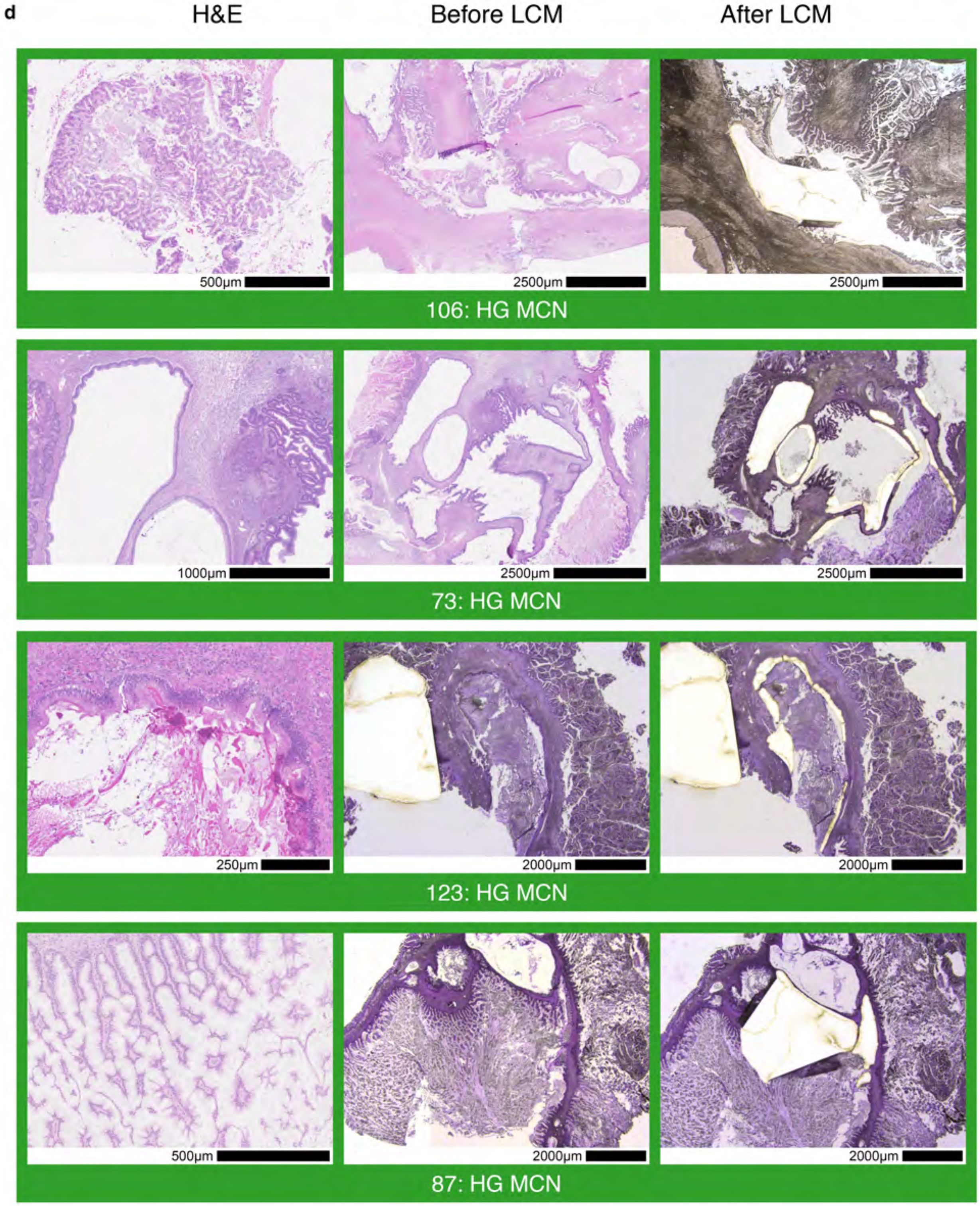

Supplementary Figure 3. Somatic mutations, phylogeny and laser capture microdissection in MTP3. 3d. Representative images of neoplastic tissue stained by hematoxylin and eosin (H&E), as well as isolated regions before and after laser capture microdissection are shown.

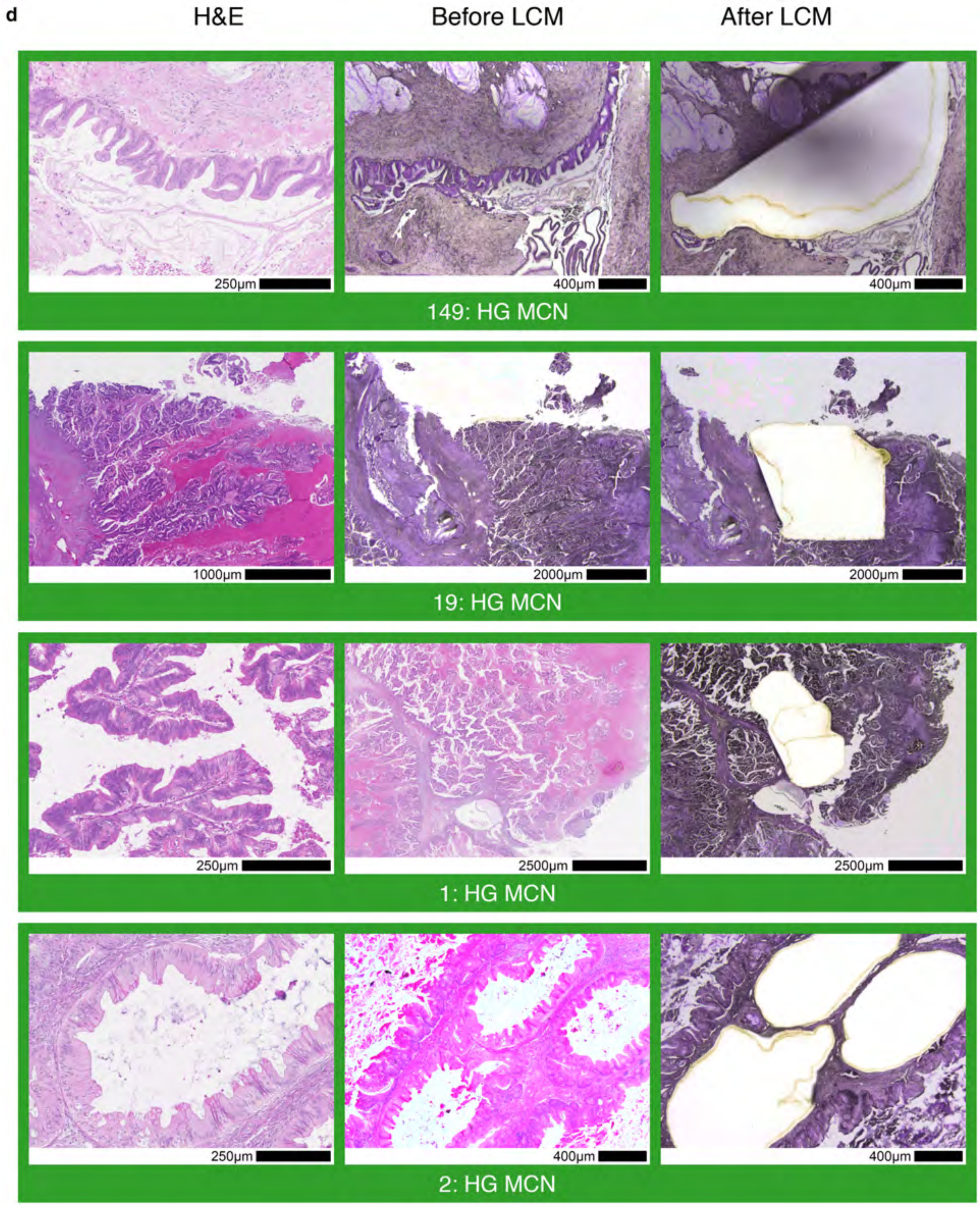

Supplementary Figure 3. Somatic mutations, phylogeny and laser capture microdissection in MTP3. 3d. Representative images of neoplastic tissue stained by hematoxylin and eosin (H&E), as well as isolated regions before and after laser capture microdissection are shown.

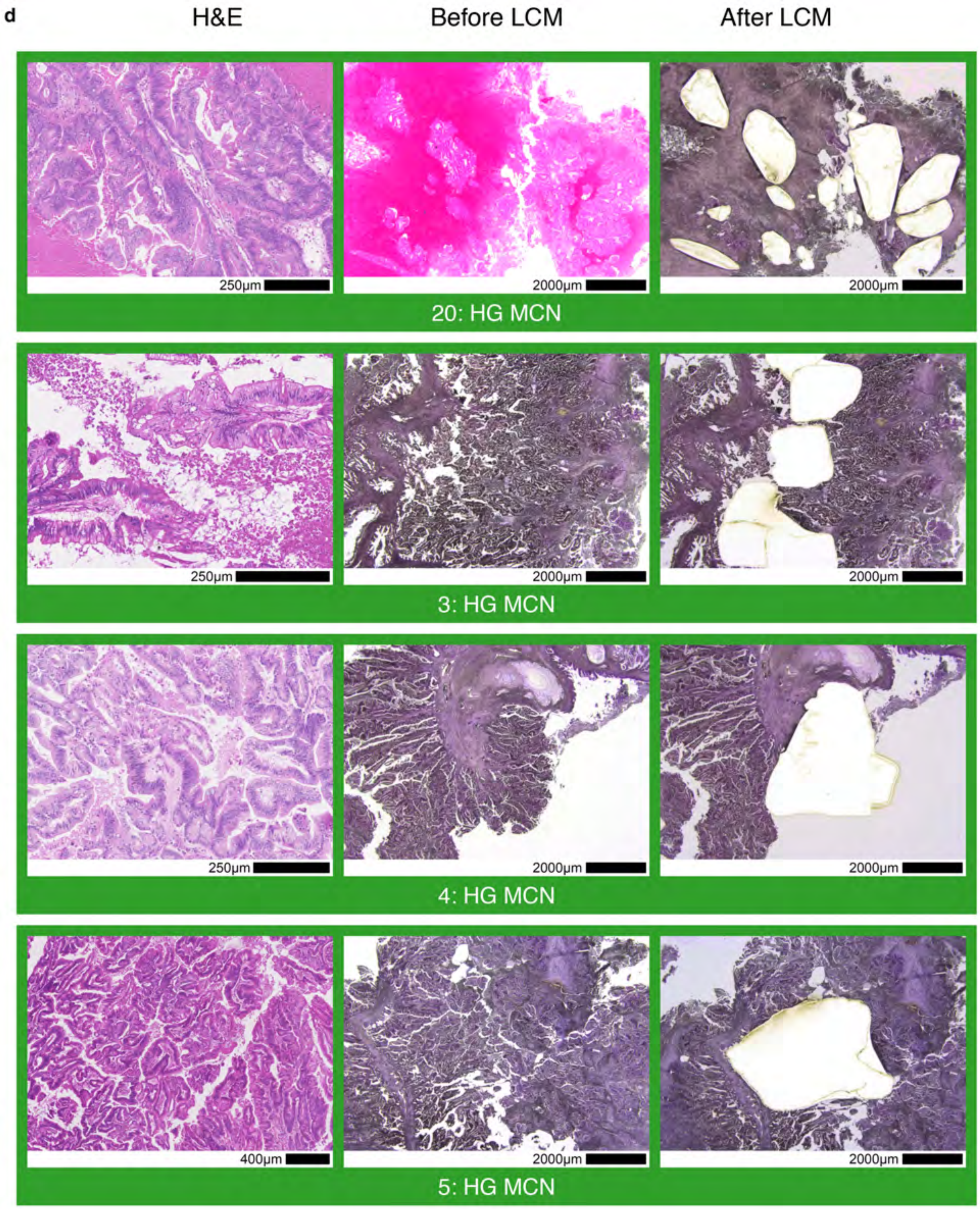

Supplementary Figure 3. Somatic mutations, phylogeny and laser capture microdissection in MTP3. 3d. Representative images of neoplastic tissue stained by hematoxylin and eosin (H&E), as well as isolated regions before and after laser capture microdissection are shown.

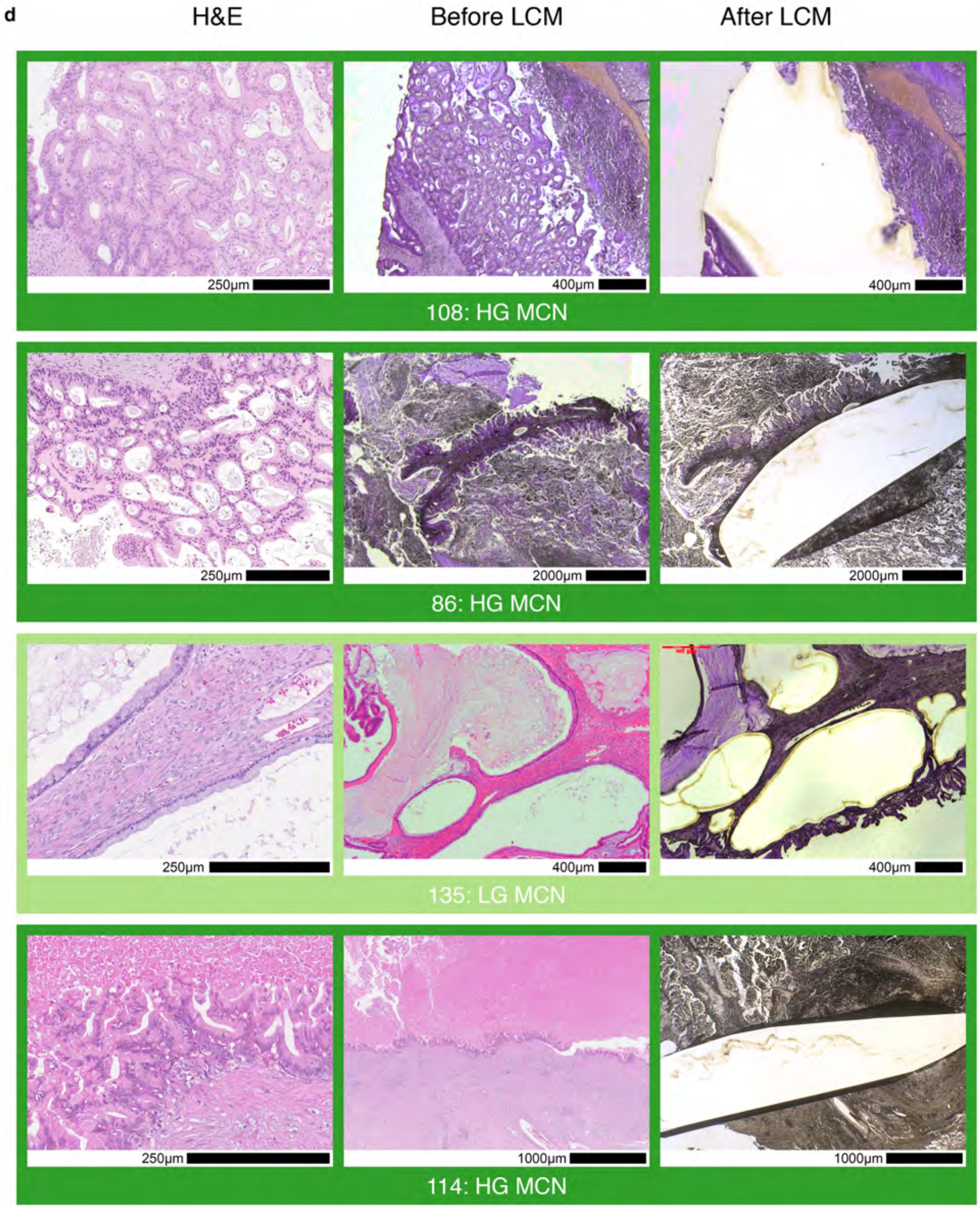

Supplementary Figure 3. Somatic mutations, phylogeny and laser capture microdissection in MTP3. 3d. Representative images of neoplastic tissue stained by hematoxylin and eosin (H&E), as well as isolated regions before and after laser capture microdissection are shown.

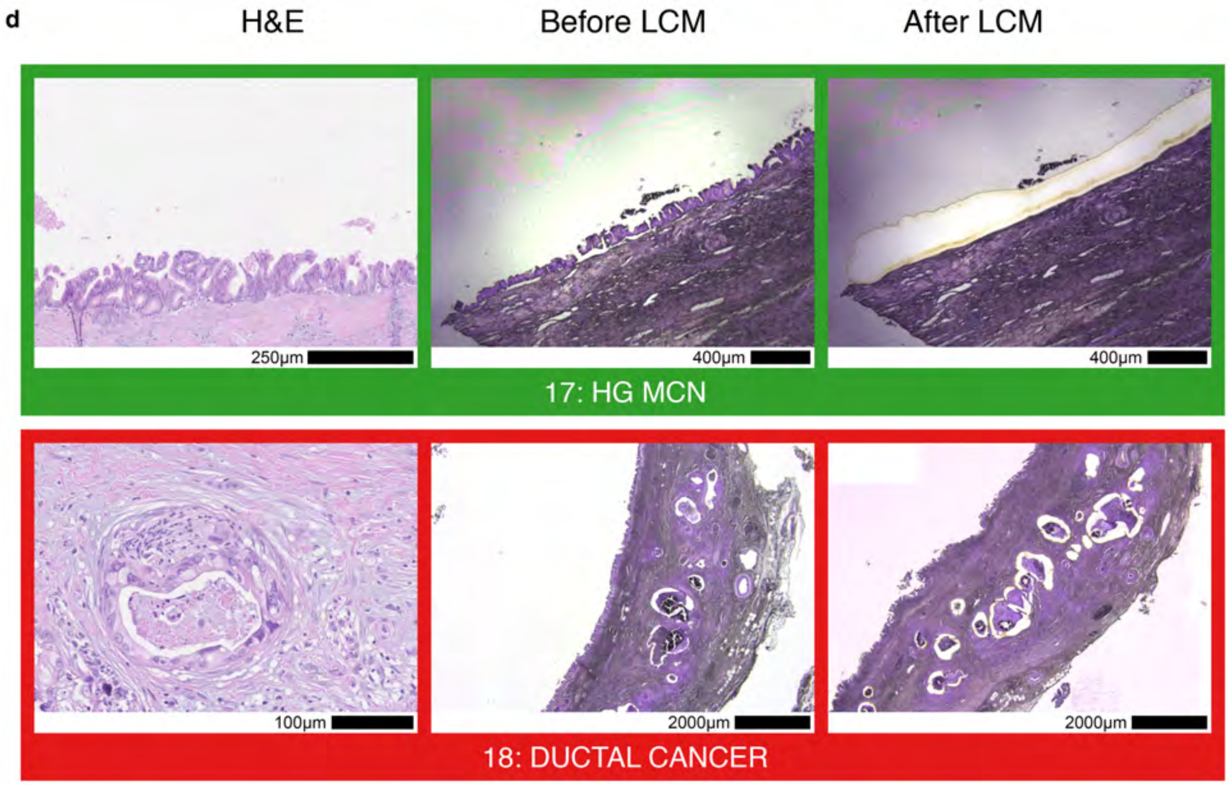

Supplementary Figure 4. Somatic mutations, phylogeny and laser capture microdissection in MTP4. 4a. Mutations (rows) identified in the different samples (columns) in MTP4. Sample and mutation characteristics are described by the legend. The type of sequencing analysis (targeted or whole exome sequencing) performed for each sample is indicated in a track on the bottom. 4b. The inferred tumor phylogeny. The pathological characteristics for the clones are indicated by the color of the line and driver mutations are indicated at branch points. 4c. Comparison of variant allele frequencies (VAFs) in sample pairs. Each plot shows the VAFs of the corresponding samples indicated on the horizontal and vertical axes. Sample and mutation types are indicated by the colors in the legend. High VAFs of mutations shared in IPMN/MCN and cancer samples demonstrate clonal relatedness and exclude the possibility of contamination by minute amounts of cells.

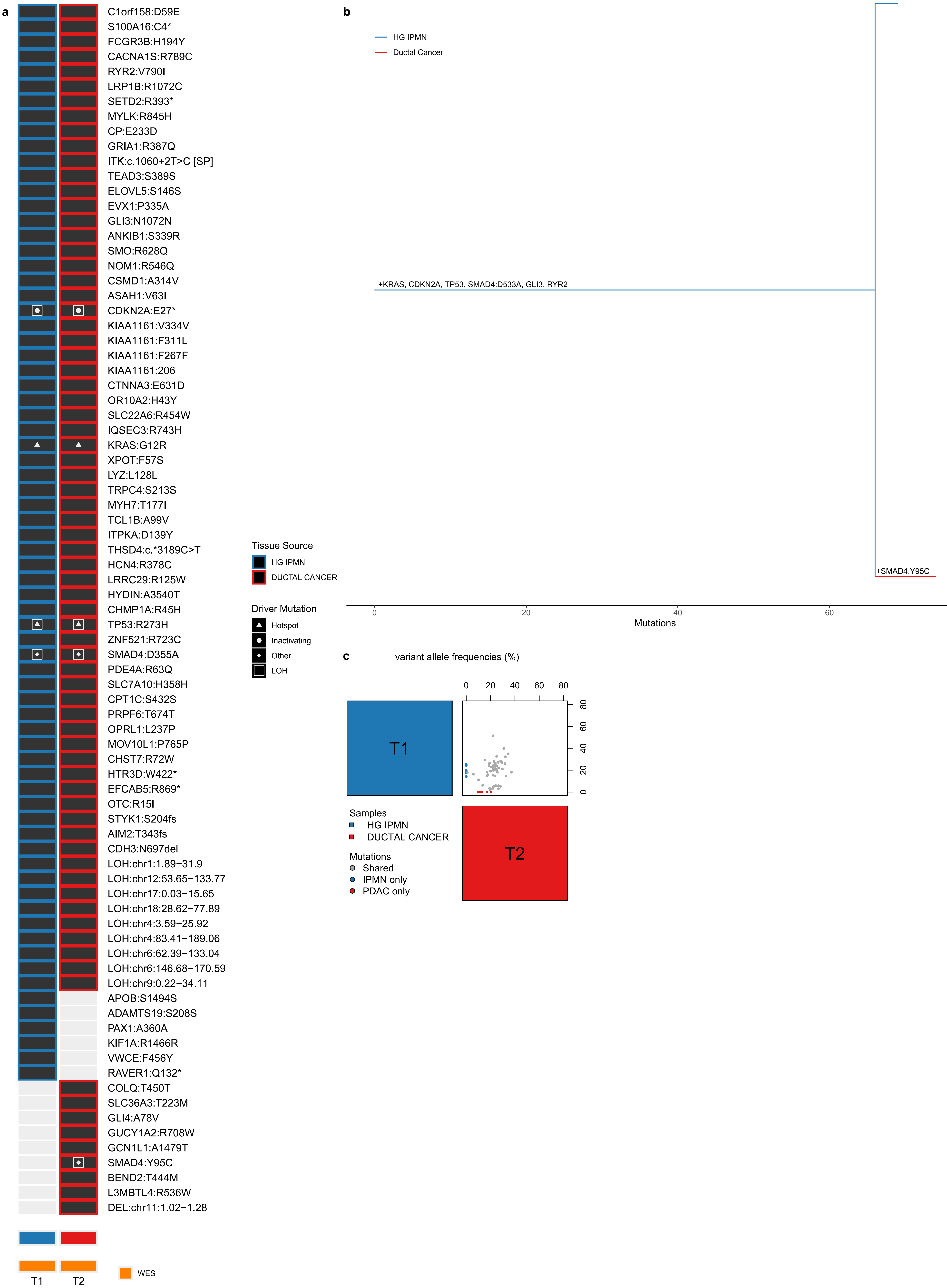

Supplementary Figure 5. Somatic mutations, phylogeny and laser capture microdissection in MTP5. 5a. Mutations (rows) identified in the different samples (columns) in MTP5. Sample and mutation characteristics are described by the legend. The type of sequencing analysis (targeted or whole exome sequencing) performed for each sample is indicated in a track on the bottom. 5b. The inferred tumor phylogeny. The pathological characteristics for the clones are indicated by the color of the line and driver mutations are indicated at branch points. 5c. Comparison of variant allele frequencies (VAFs) in sample pairs. Each plot shows the VAFs of the corresponding samples indicated on the horizontal and vertical axes. Sample and mutation types are indicated by the colors in the legend. High VAFs of mutations shared in IPMN/MCN and cancer samples demonstrate clonal relatedness and exclude the possibility of contamination by minute amounts of cells.

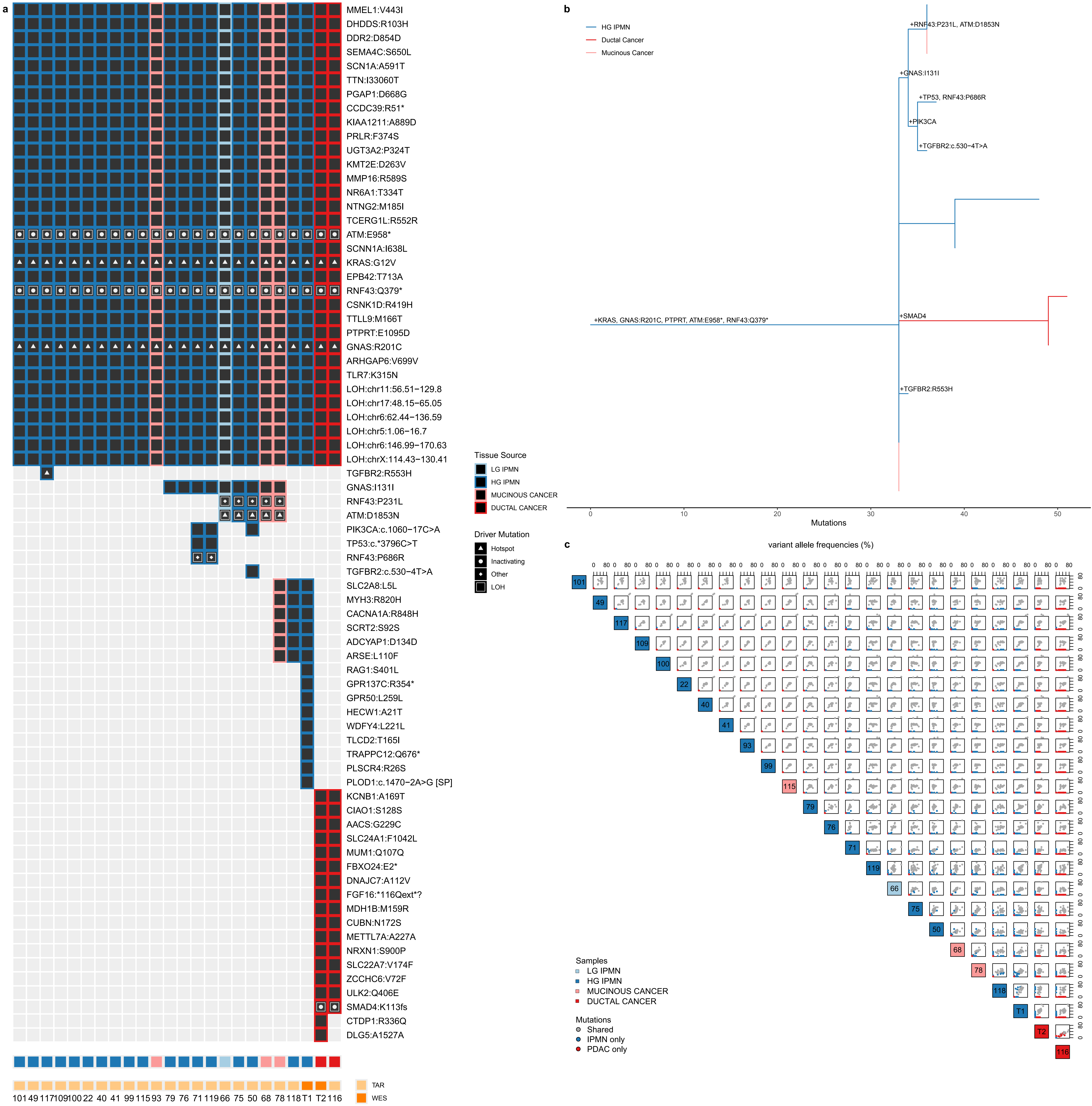

Supplementary Figure 5. Somatic mutations, phylogeny and laser capture microdissection in MTP5. 5d. Representative images of neoplastic tissue stained by hematoxylin and eosin (H&E), as well as isolated regions before and after laser capture microdissection are shown.

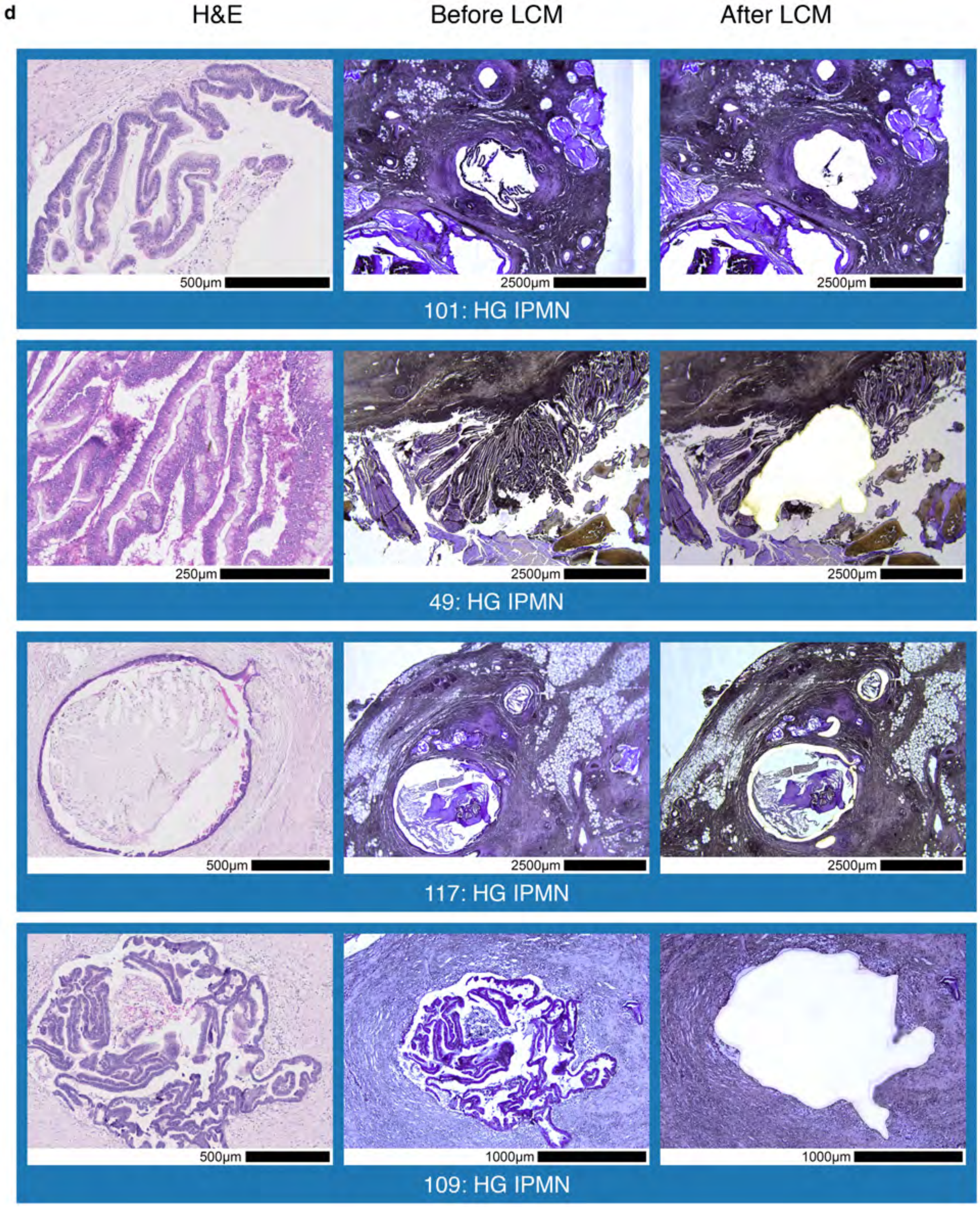

Supplementary Figure 5. Somatic mutations, phylogeny and laser capture microdissection in MTP5. 5d. Representative images of neoplastic tissue stained by hematoxylin and eosin (H&E), as well as isolated regions before and after laser capture microdissection are shown.

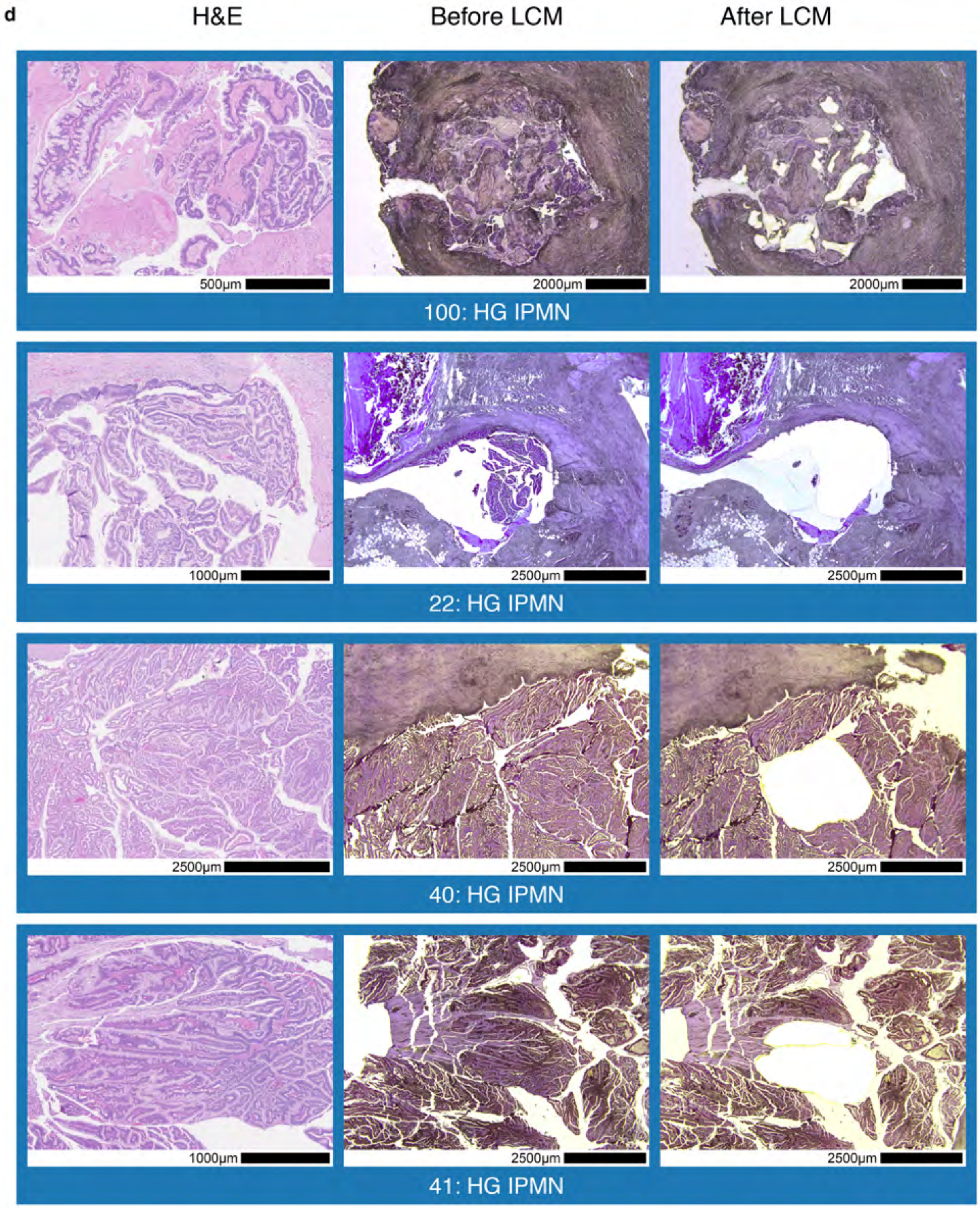

Supplementary Figure 5. Somatic mutations, phylogeny and laser capture microdissection in MTP5. 5d. Representative images of neoplastic tissue stained by hematoxylin and eosin (H&E), as well as isolated regions before and after laser capture microdissection are shown.

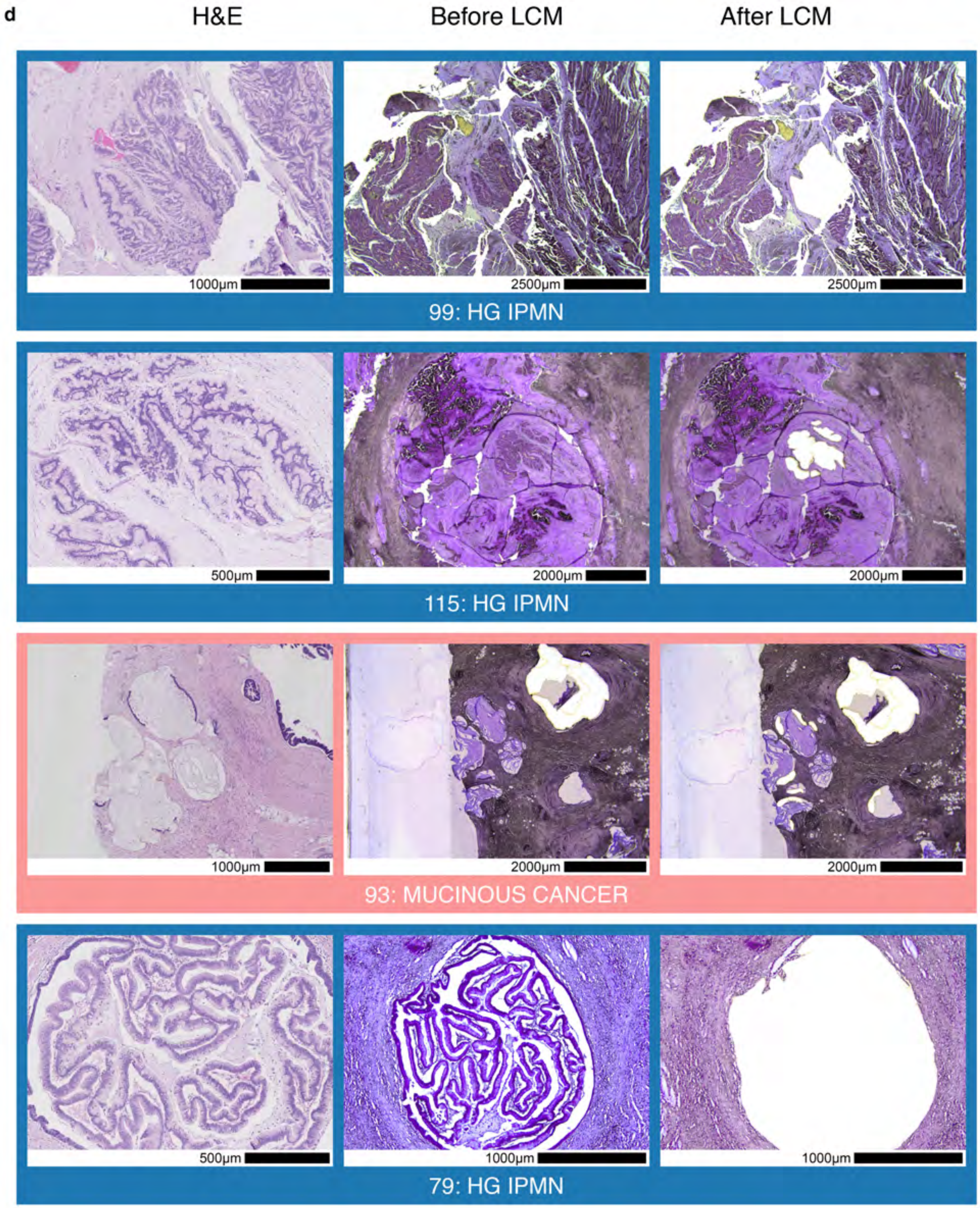

Supplementary Figure 5. Somatic mutations, phylogeny and laser capture microdissection in MTP5. 5d. Representative images of neoplastic tissue stained by hematoxylin and eosin (H&E), as well as isolated regions before and after laser capture microdissection are shown.

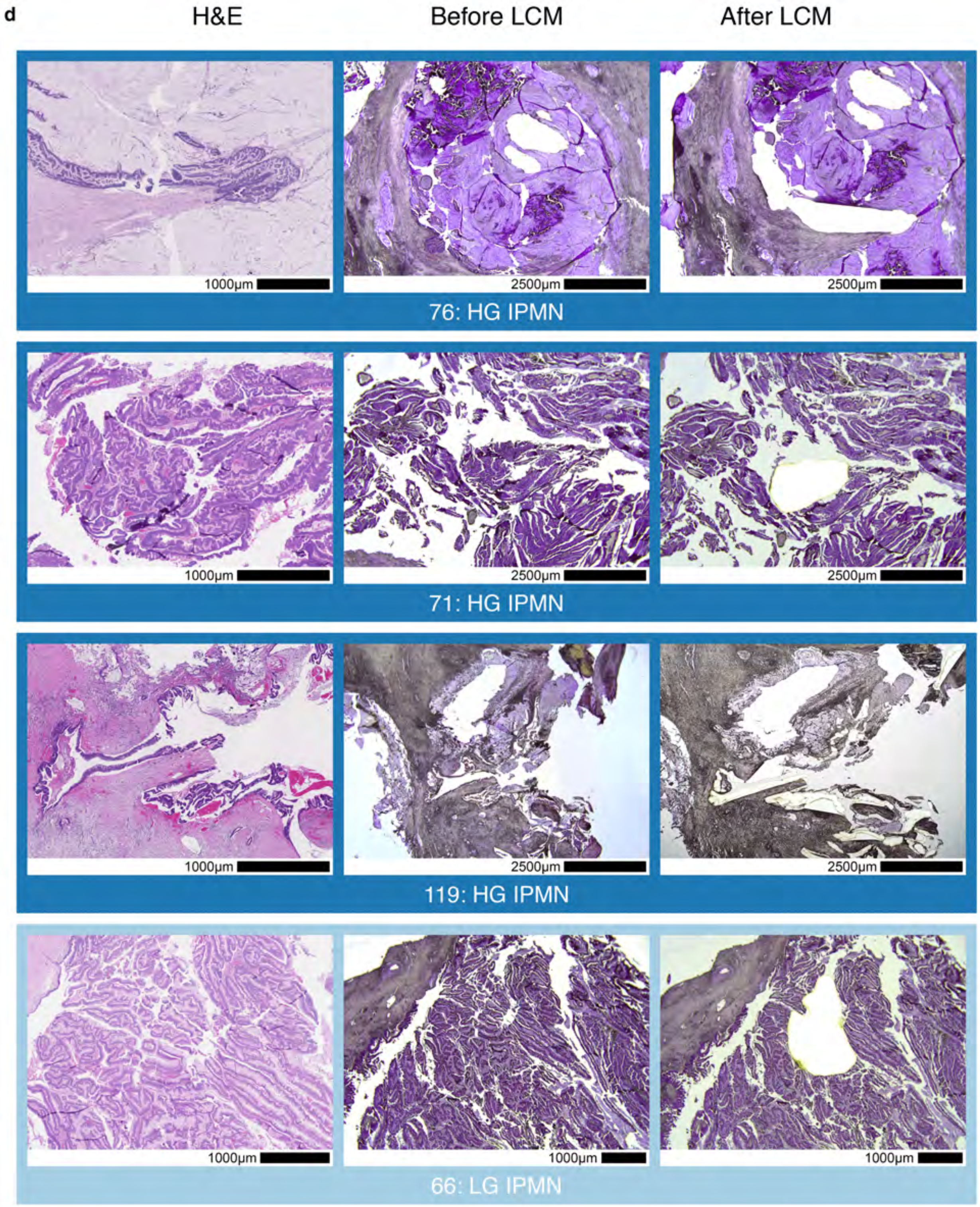

Supplementary Figure 5. Somatic mutations, phylogeny and laser capture microdissection in MTP5. 5d. Representative images of neoplastic tissue stained by hematoxylin and eosin (H&E), as well as isolated regions before and after laser capture microdissection are shown.

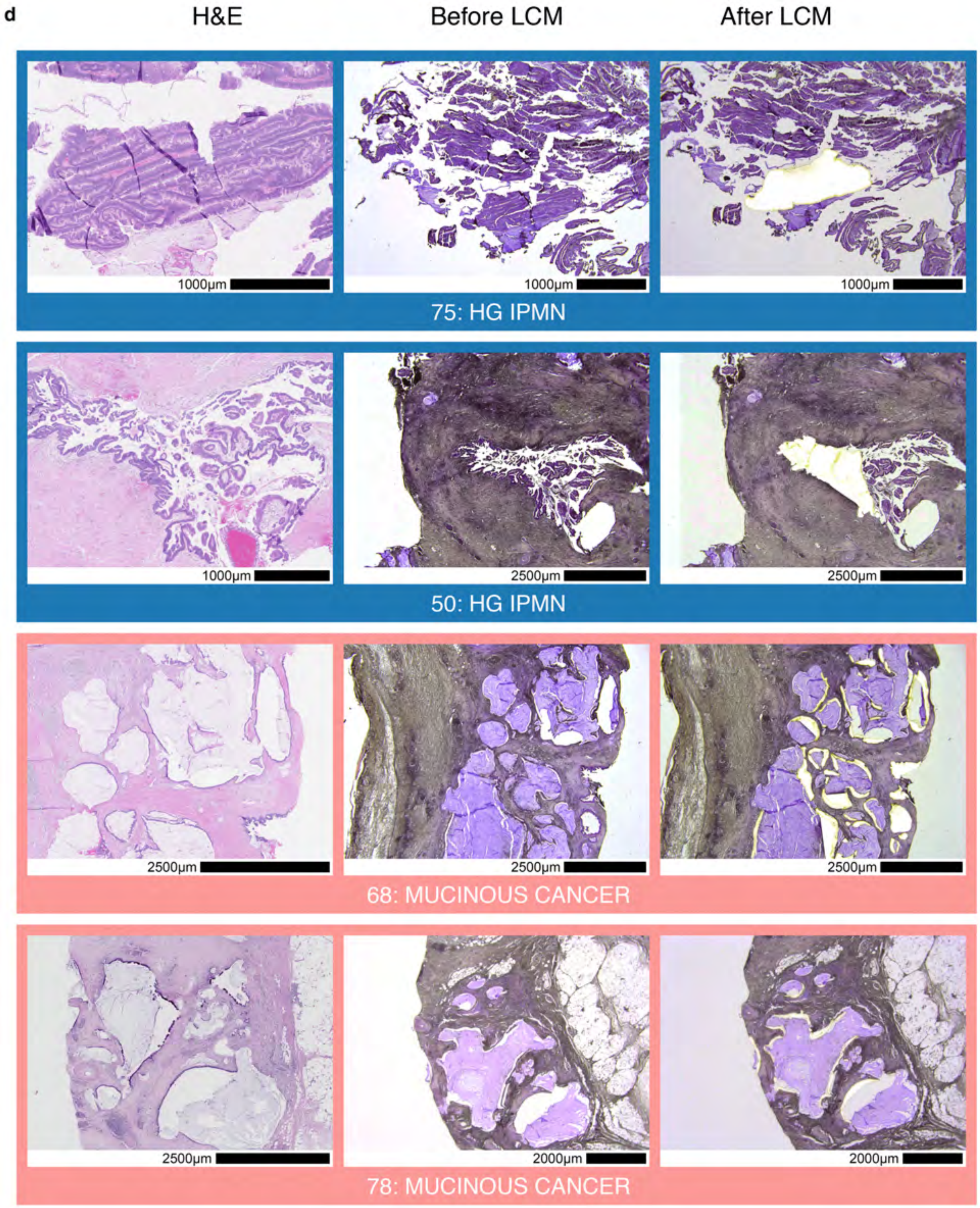

Supplementary Figure 5. Somatic mutations, phylogeny and laser capture microdissection in MTP5. 5d. Representative images of neoplastic tissue stained by hematoxylin and eosin (H&E), as well as isolated regions before and after laser capture microdissection are shown.

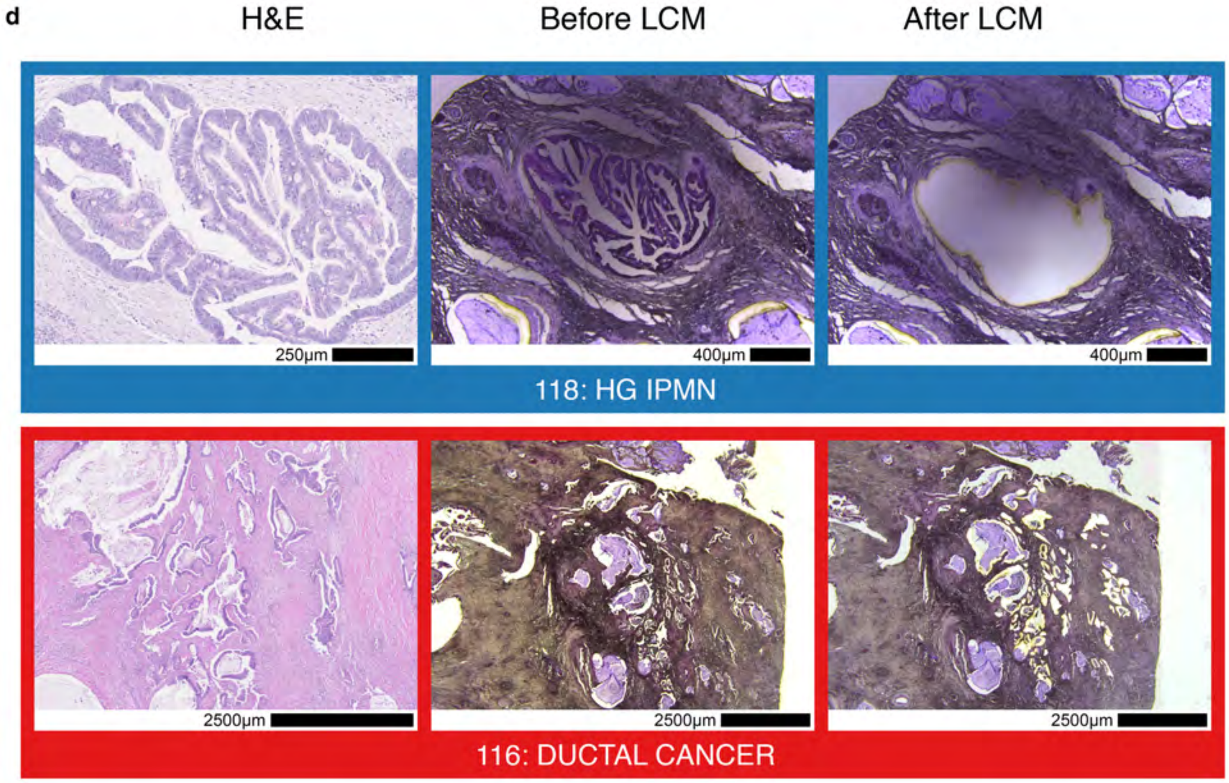

Supplementary Figure 6. Somatic mutations and phylogeny in MTP6. 6a. Mutations (rows) identified in the different samples (columns) in MTP6. Sample and mutation characteristics are described by the legend. The type of sequencing analysis (targeted or whole exome sequencing) performed for each sample is indicated in a track on the bottom. 6b. The inferred tumor phylogeny. The pathological characteristics for the clones are indicated by the color of the line and driver mutations are indicated at branch points. 6c. Comparison of variant allele frequencies (VAFs) in precancer/cancer sample pair. Sample and mutation types are indicated by the colors in the legend. High VAFs of mutations shared in IPMN/MCN and cancer samples demonstrate clonal relatedness and exclude the possibility of contamination by minute amounts of cells.

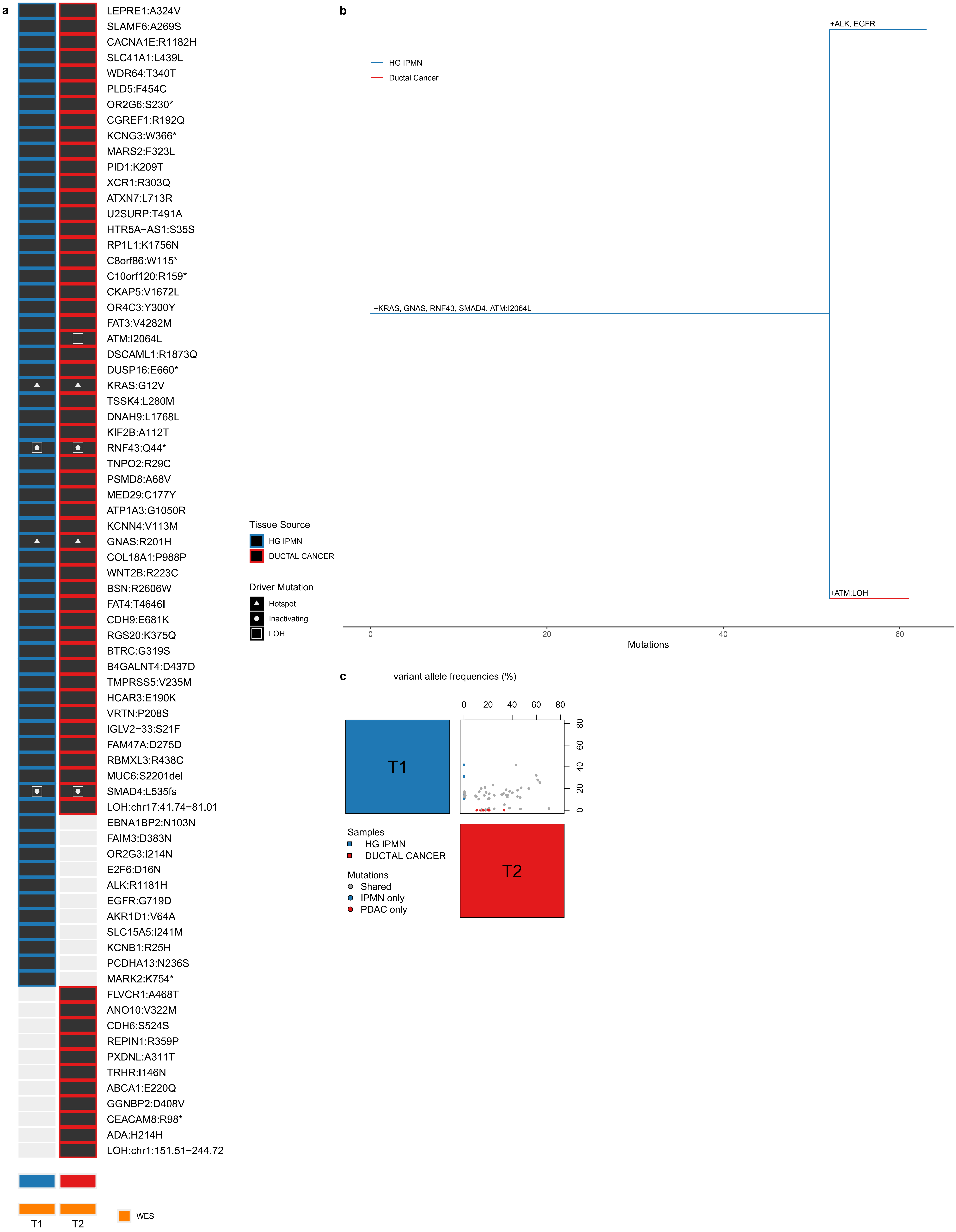

Supplementary Figure 7. Somatic mutations and phylogeny in MTP7. 7a. Mutations (rows) identified in the different samples (columns) in MTP7. Sample and mutation characteristics are described by the legend. The type of sequencing analysis (targeted or whole exome sequencing) performed for each sample is indicated in a track on the bottom. 7b. The inferred tumor phylogeny. The pathological characteristics for the clones are indicated by the color of the line and driver mutations are indicated at branch points. 7c. Comparison of variant allele frequencies (VAFs) in precancer/cancer sample pair. Sample and mutation types are indicated by the colors in the legend. High VAFs of mutations shared in IPMN/MCN and cancer samples demonstrate clonal relatedness and exclude the possibility of contamination by minute amounts of cells.

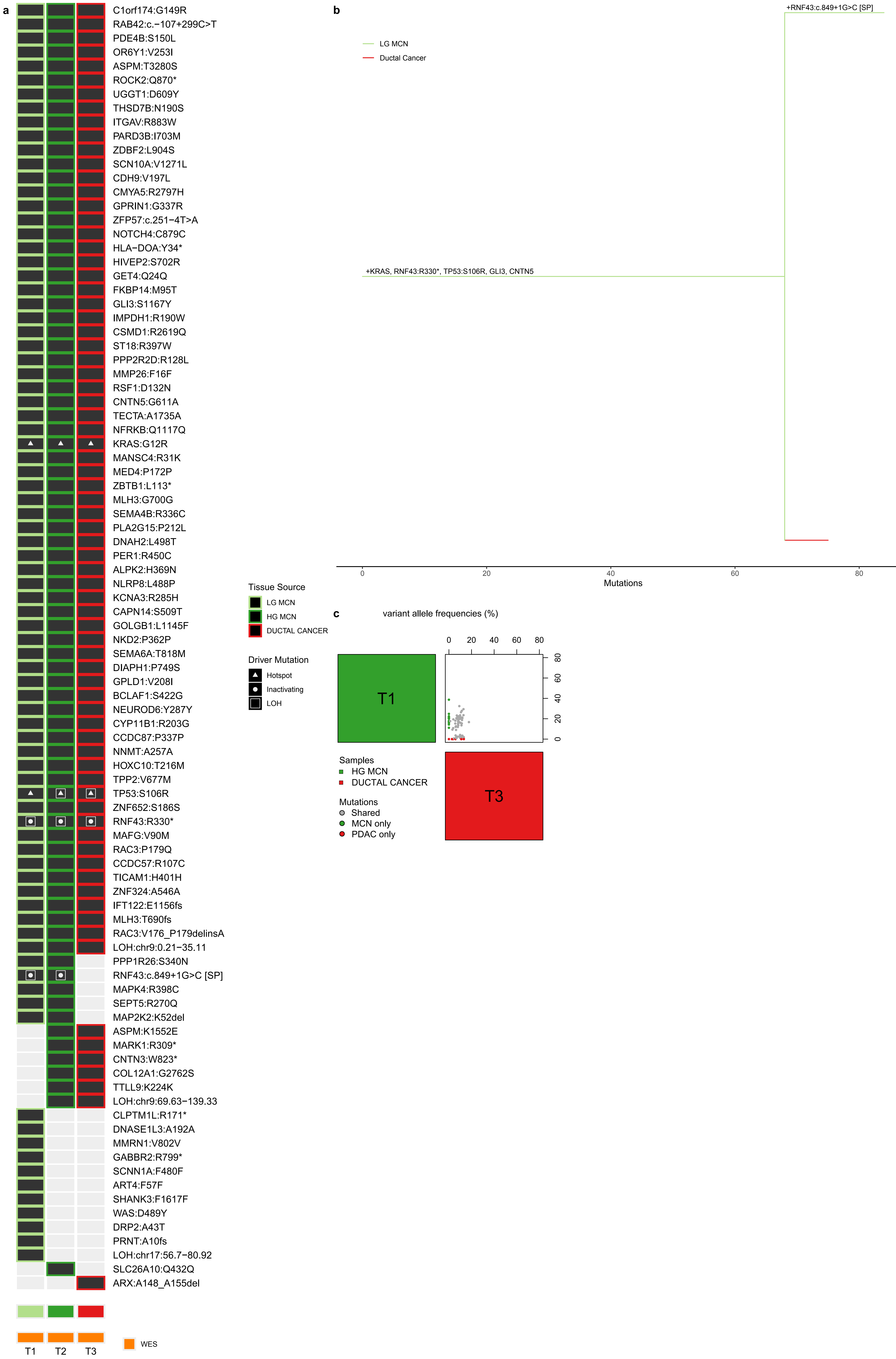

Supplementary Figure 8. Somatic mutations, phylogeny and laser capture microdissection in MTP8. 8a. Mutations (rows) identified in the different samples (columns) in MTP8. Sample and mutation characteristics are described by the legend. The type of sequencing analysis (targeted or whole exome sequencing) performed for each sample is indicated in a track on the bottom. 8b. The inferred tumor phylogeny. The pathological characteristics for the clones are indicated by the color of the line and driver mutations are indicated at branch points. 8c. Comparison of variant allele frequencies (VAFs) in sample pairs. Each plot shows the VAFs of the corresponding samples indicated on the horizontal and vertical axes. Sample and mutation types are indicated by the colors in the legend. High VAFs of mutations shared in IPMN/MCN and cancer samples demonstrate clonal relatedness and exclude the possibility of contamination by minute amounts of cells.

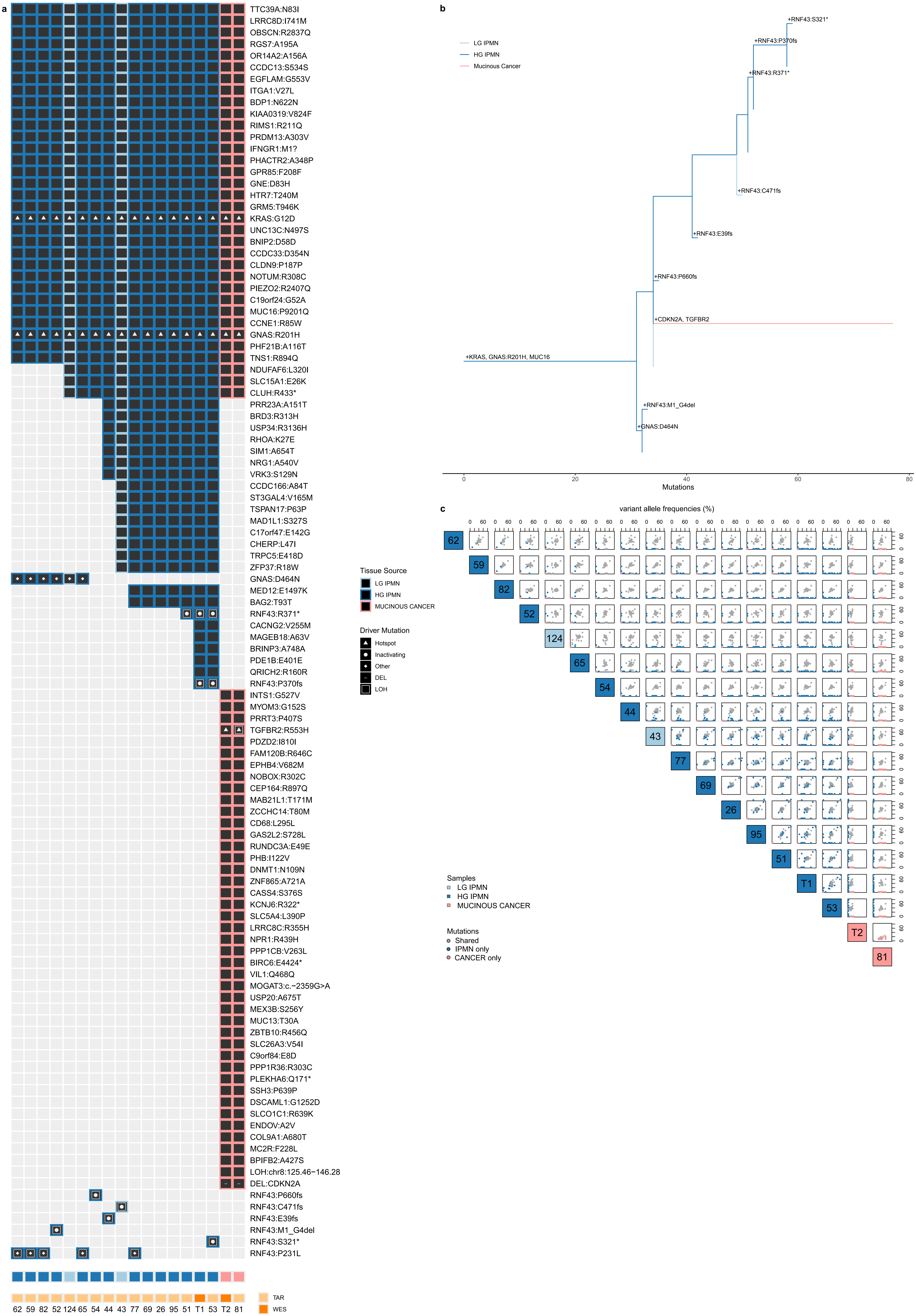

Supplementary Figure 8. Somatic mutations, phylogeny and laser capture microdissection in MTP8. 8d. Representative images of neoplastic tissue stained by hematoxylin and eosin (H&E), as well as isolated regions before and after laser capture microdissection are shown.

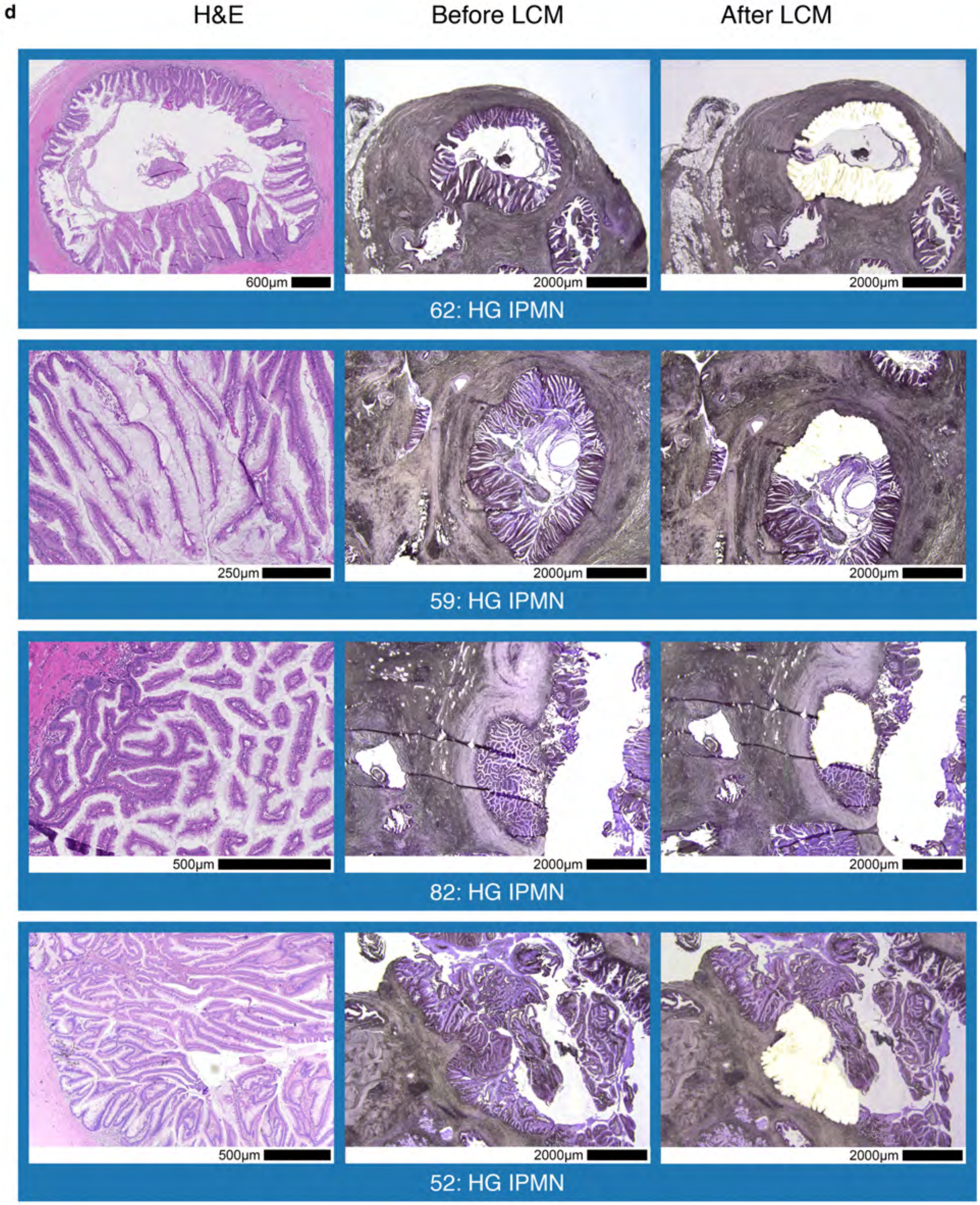

Supplementary Figure 8. Somatic mutations, phylogeny and laser capture microdissection in MTP8. 8d. Representative images of neoplastic tissue stained by hematoxylin and eosin (H&E), as well as isolated regions before and after laser capture microdissection are shown.

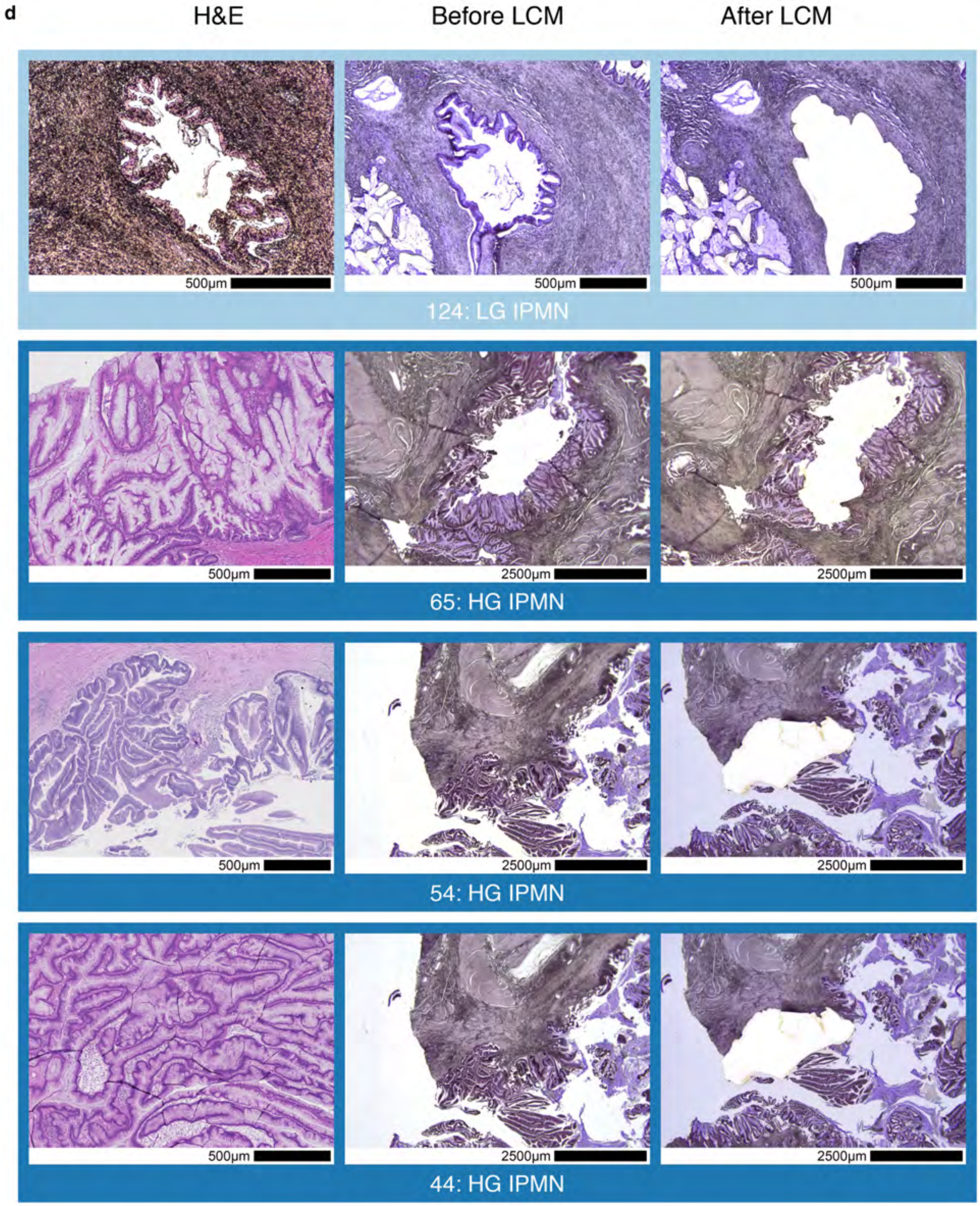

Supplementary Figure 8. Somatic mutations, phylogeny and laser capture microdissection in MTP8. 8d. Representative images of neoplastic tissue stained by hematoxylin and eosin (H&E), as well as isolated regions before and after laser capture microdissection are shown.

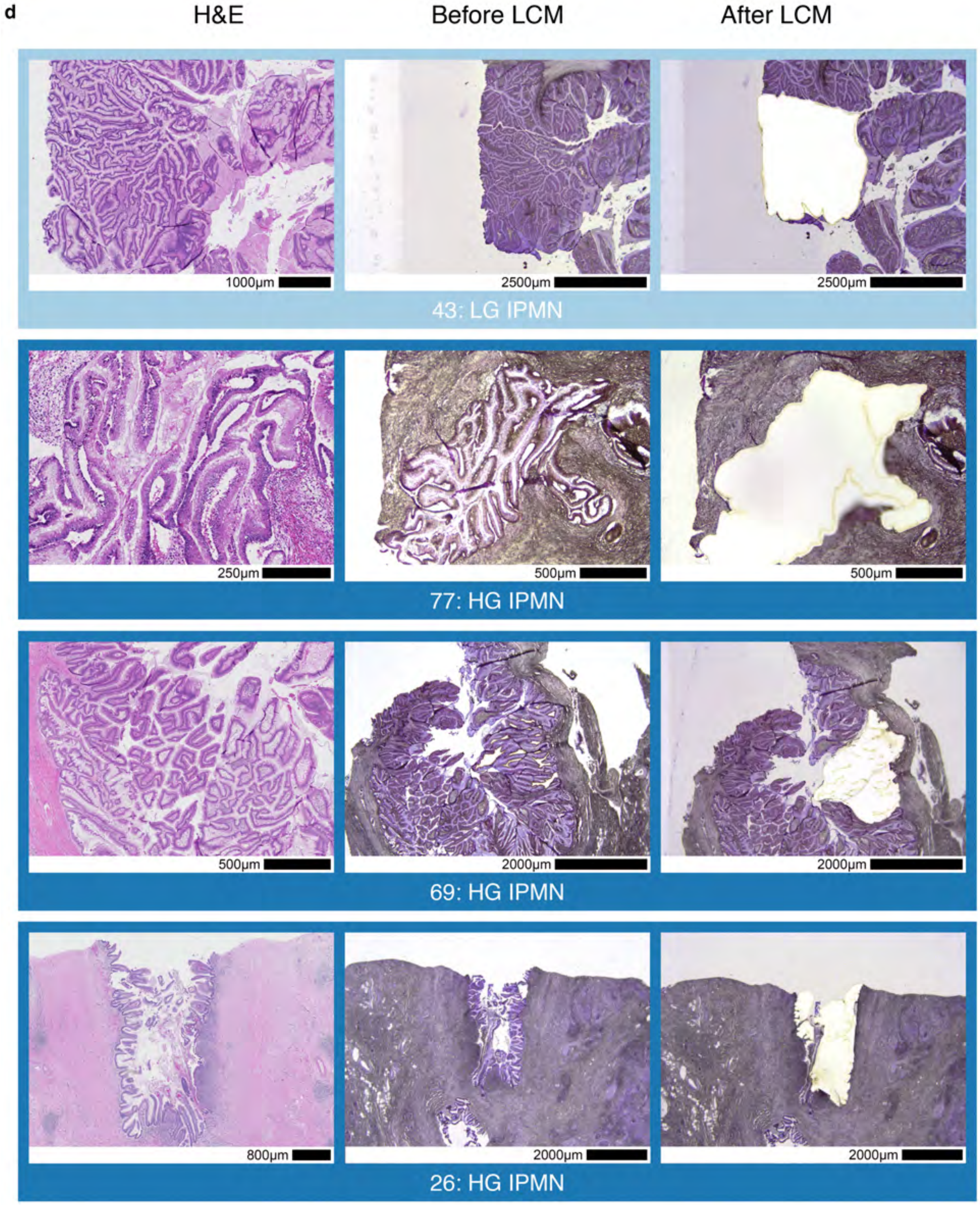

Supplementary Figure 8. Somatic mutations, phylogeny and laser capture microdissection in MTP8. 8d. Representative images of neoplastic tissue stained by hematoxylin and eosin (H&E), as well as isolated regions before and after laser capture microdissection are shown.

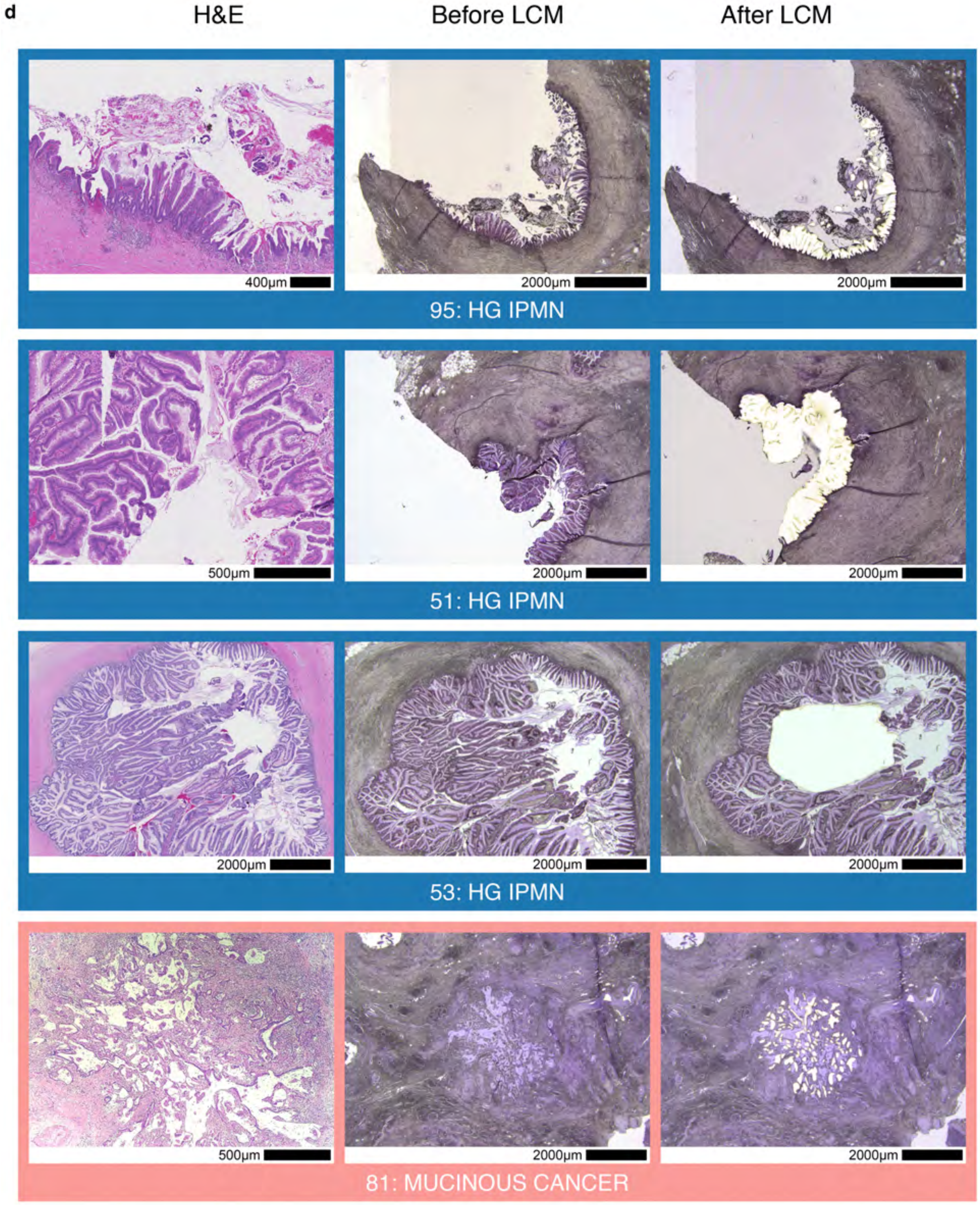

Supplementary Figure 9. Somatic mutations and phylogeny in MTP9. 9a. Mutations (rows) identified in the different samples (columns) in MTP9. Sample and mutation characteristics are described by the legend. The type of sequencing analysis (targeted or whole exome sequencing) performed for each sample is indicated in a track on the bottom. 9b. The inferred tumor phylogeny. The pathological characteristics for the clones are indicated by the color of the line and driver mutations are indicated at branch points. 9c. Comparison of variant allele frequencies (VAFs) in precancer/cancer sample pair. Sample and mutation types are indicated by the colors in the legend. High VAFs of mutations shared in IPMN/MCN and cancer samples demonstrate clonal relatedness and exclude the possibility of contamination by minute amounts of cells.

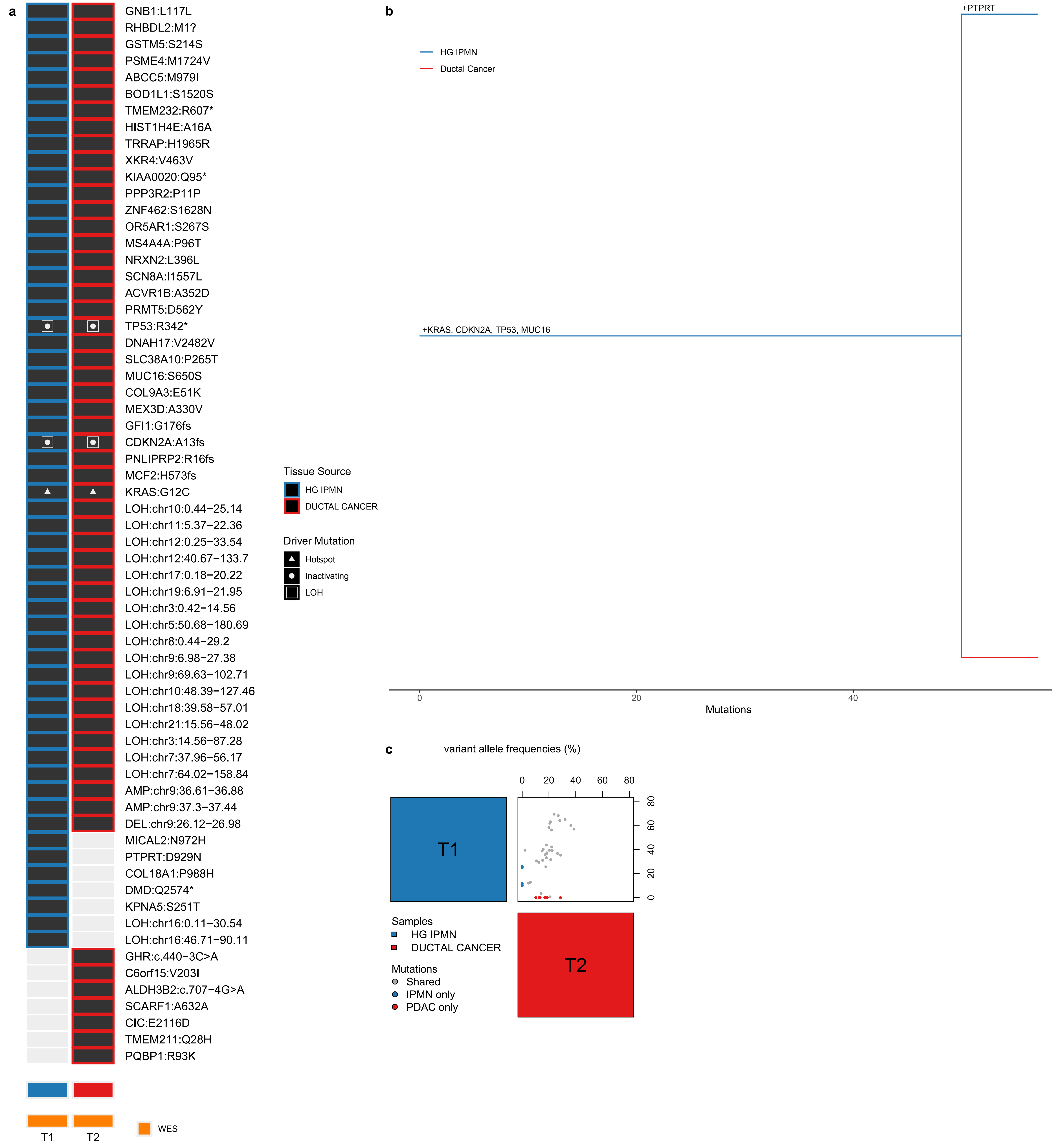

Supplementary Figure 10. Somatic mutations and phylogeny in MTP11. 10a. Mutations (rows) identified in the different samples (columns) in MTP11. Sample and mutation characteristics are described by the legend. The type of sequencing analysis (targeted or whole exome sequencing) performed for each sample is indicated in a track on the bottom. 10b. The inferred tumor phylogeny. The pathological characteristics for the clones are indicated by the color of the line and driver mutations are indicated at branch points. 10c. Comparison of variant allele frequencies (VAFs) in precancer/cancer sample pair. Sample and mutation types are indicated by the colors in the legend. High VAFs of mutations shared in IPMN/MCN and cancer samples demonstrate clonal relatedness and exclude the possibility of contamination by minute amounts of cells.

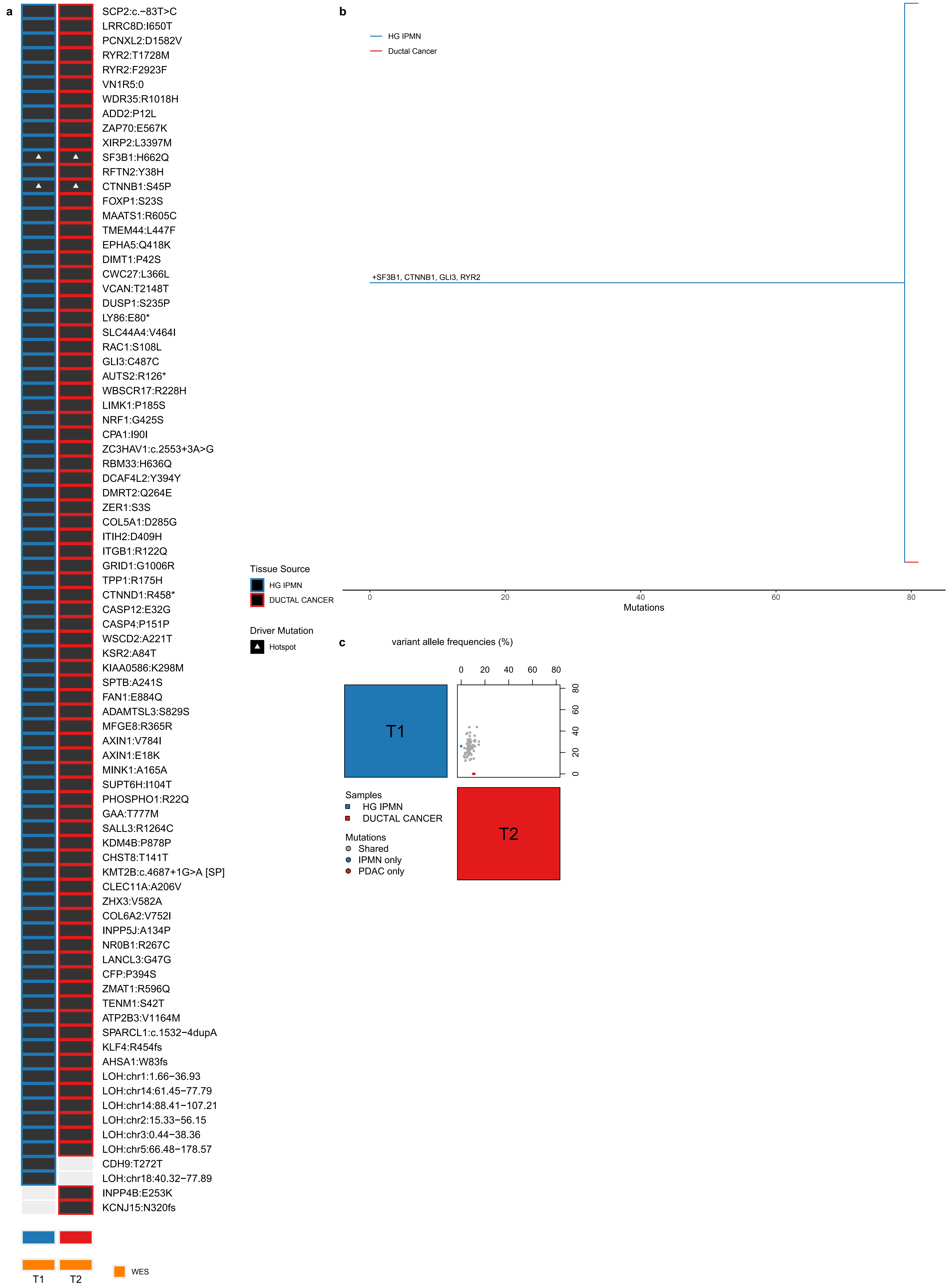

Supplementary Figure 11. Somatic mutations and phylogeny in MTP13. 11a. Mutations (rows) identified in the different samples (columns) in MTP13. Sample and mutation characteristics are described by the legend. The type of sequencing analysis (targeted or whole exome sequencing) performed for each sample is indicated in a track on the bottom. 11b. The inferred tumor phylogeny. The pathological characteristics for the clones are indicated by the color of the line and driver mutations are indicated at branch points. 11c. Comparison of variant allele frequencies (VAFs) in precancer/cancer sample pair. Sample and mutation types are indicated by the colors in the legend. High VAFs of mutations shared in IPMN/MCN and cancer samples demonstrate clonal relatedness and exclude the possibility of contamination by minute amounts of cells.

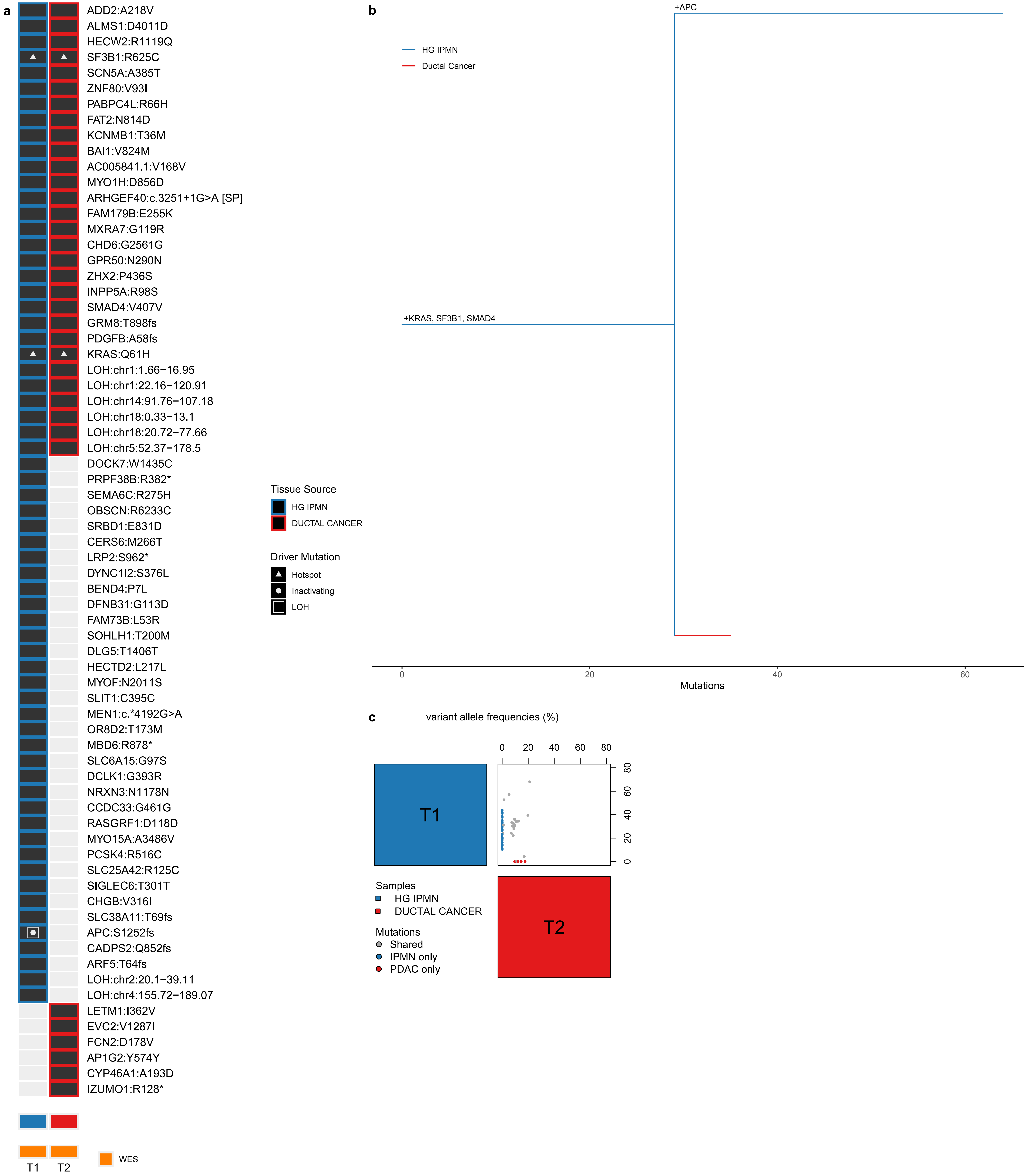

Supplementary Figure 12. Somatic mutations and phylogeny in MTP14. 12a. Mutations (rows) identified in the different samples (columns) in MTP14. Sample and mutation characteristics are described by the legend. The type of sequencing analysis (targeted or whole exome sequencing) performed for each sample is indicated in a track on the bottom. 12b. The inferred tumor phylogeny. The pathological characteristics for the clones are indicated by the color of the line and driver mutations are indicated at branch points. 12c. Comparison of variant allele frequencies (VAFs) in precancer/cancer sample pair. Sample and mutation types are indicated by the colors in the legend. High VAFs of mutations shared in IPMN/MCN and cancer samples demonstrate clonal relatedness and exclude the possibility of contamination by minute amounts of cells.

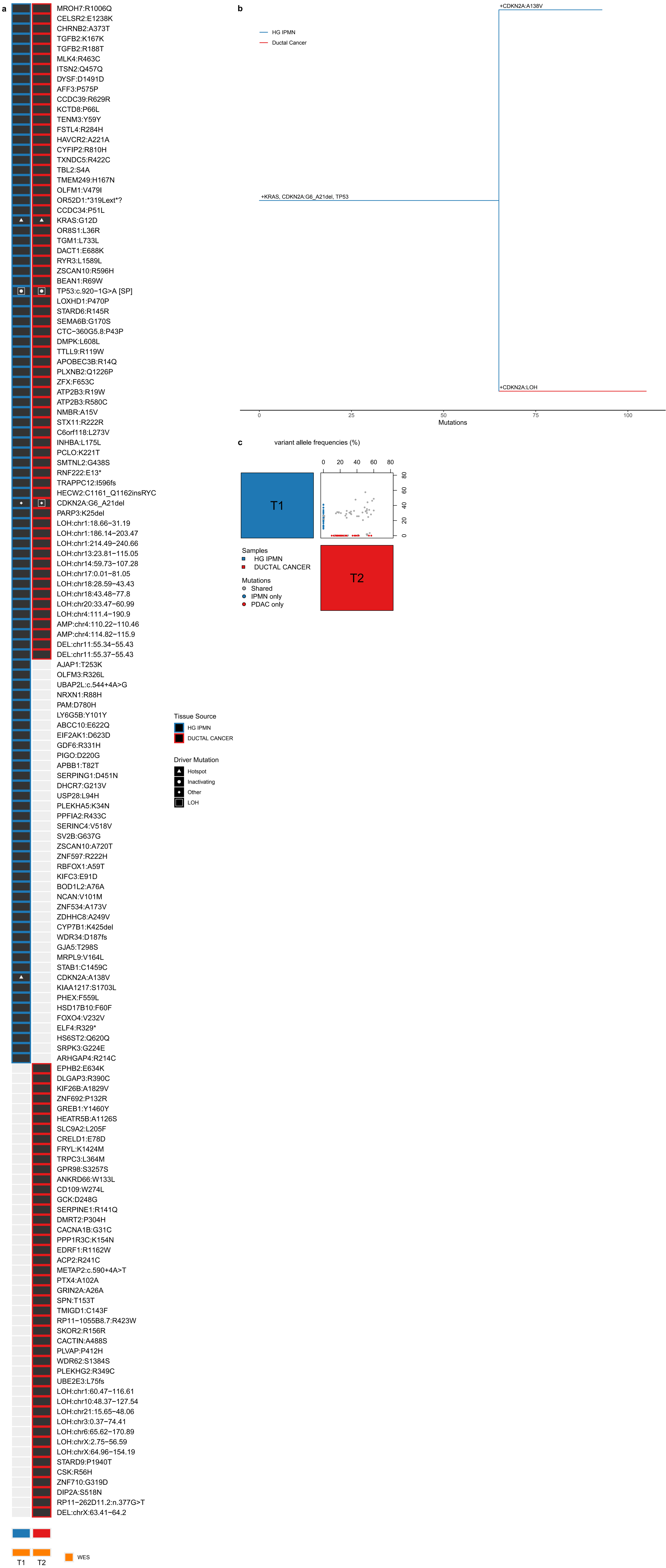

Supplementary Figure 13. Somatic mutations and phylogeny in MTP18. 13a. Mutations (rows) identified in the different samples (columns) in MTP18. Sample and mutation characteristics are described by the legend. The type of sequencing analysis (targeted or whole exome sequencing) performed for each sample is indicated in a track on the bottom. 13b. The inferred tumor phylogeny. The pathological characteristics for the clones are indicated by the color of the line and driver mutations are indicated at branch points. 13c. Comparison of variant allele frequencies (VAFs) in precancer/cancer sample pair. Sample and mutation types are indicated by the colors in the legend. High VAFs of mutations shared in IPMN/MCN and cancer samples demonstrate clonal relatedness and exclude the possibility of contamination by minute amounts of cells.

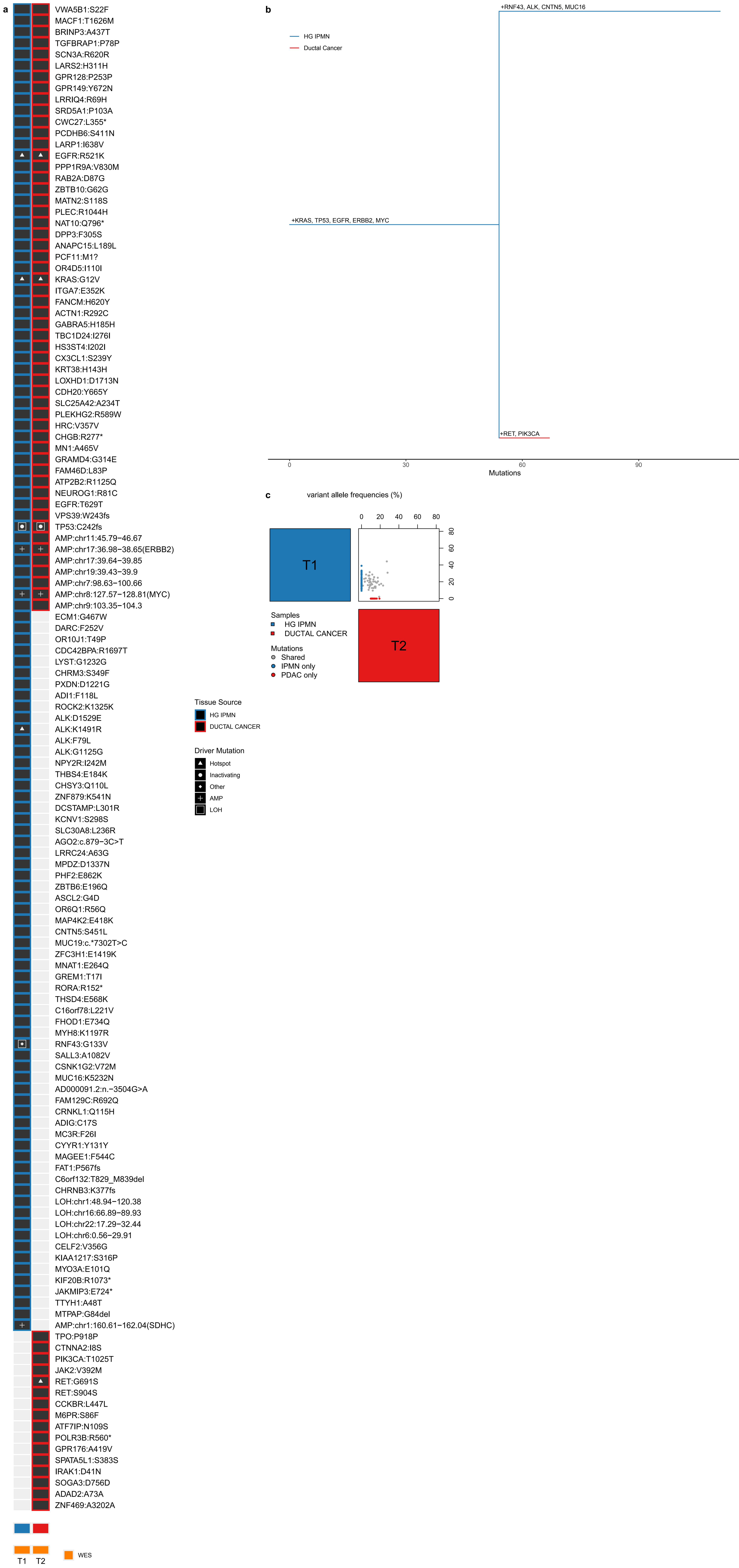

Supplementary Figure 14. Somatic mutations, phylogeny and laser capture microdissection in MTP19. 14a. Mutations (rows) identified in the different samples (columns) in MTP19. Sample and mutation characteristics are described by the legend. The type of sequencing analysis (targeted or whole exome sequencing) performed for each sample is indicated in a track on the bottom. 14b. The inferred tumor phylogeny. The pathological characteristics for the clones are indicated by the color of the line and driver mutations are indicated at branch points. 14c. Comparison of variant allele frequencies (VAFs) in sample pairs. Each plot shows the VAFs of the corresponding samples indicated on the horizontal and vertical axes. Sample and mutation types are indicated by the colors in the legend. High VAFs of mutations shared in IPMN/MCN and cancer samples demonstrate clonal relatedness and exclude the possibility of contamination by minute amounts of cells.

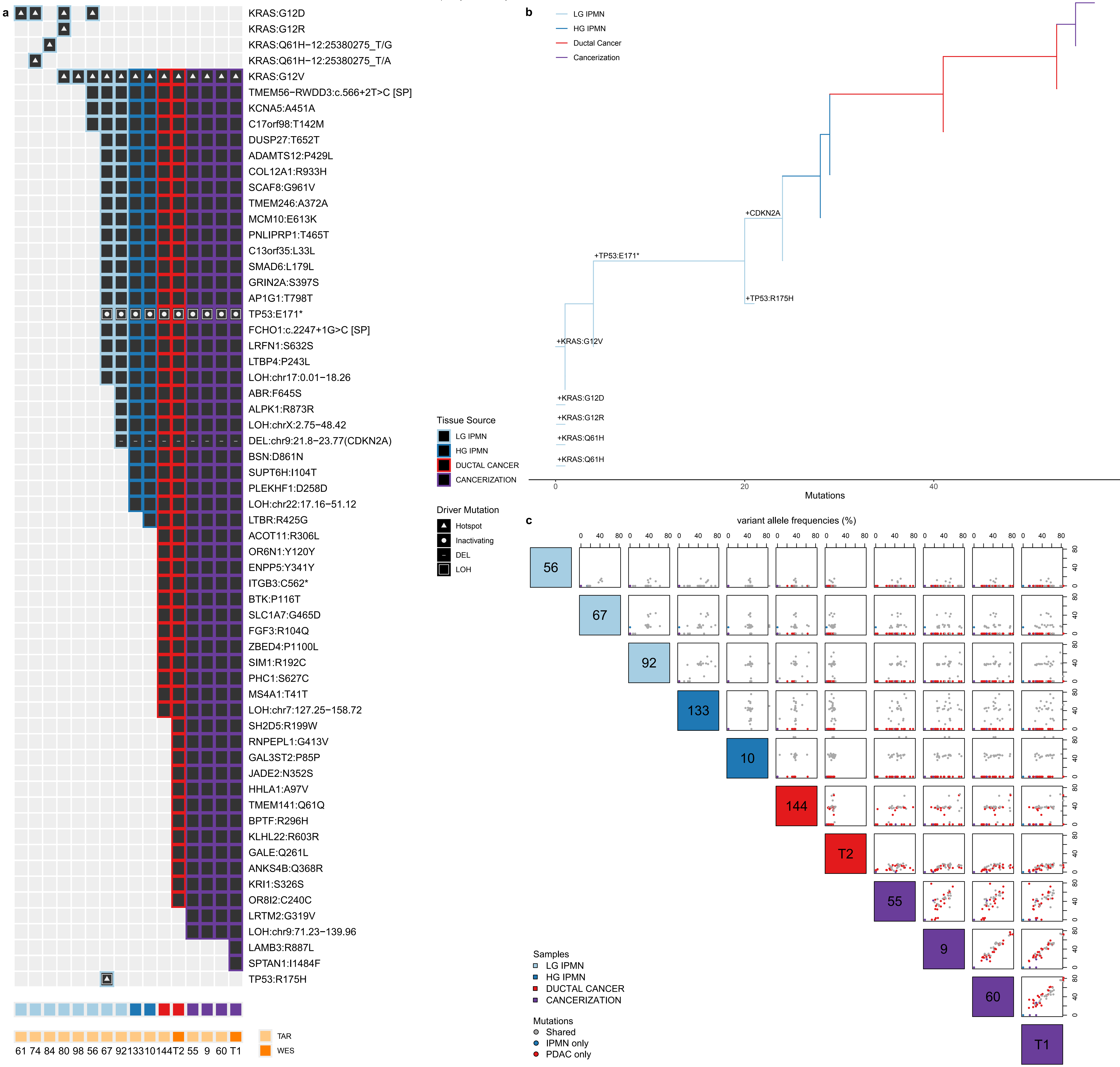

Supplementary Figure 14. Somatic mutations, phylogeny and laser capture microdissection in MTP19. 14d. Representative images of neoplastic tissue stained by hematoxylin and eosin (H&E), as well as isolated regions before and after laser capture microdissection are shown.

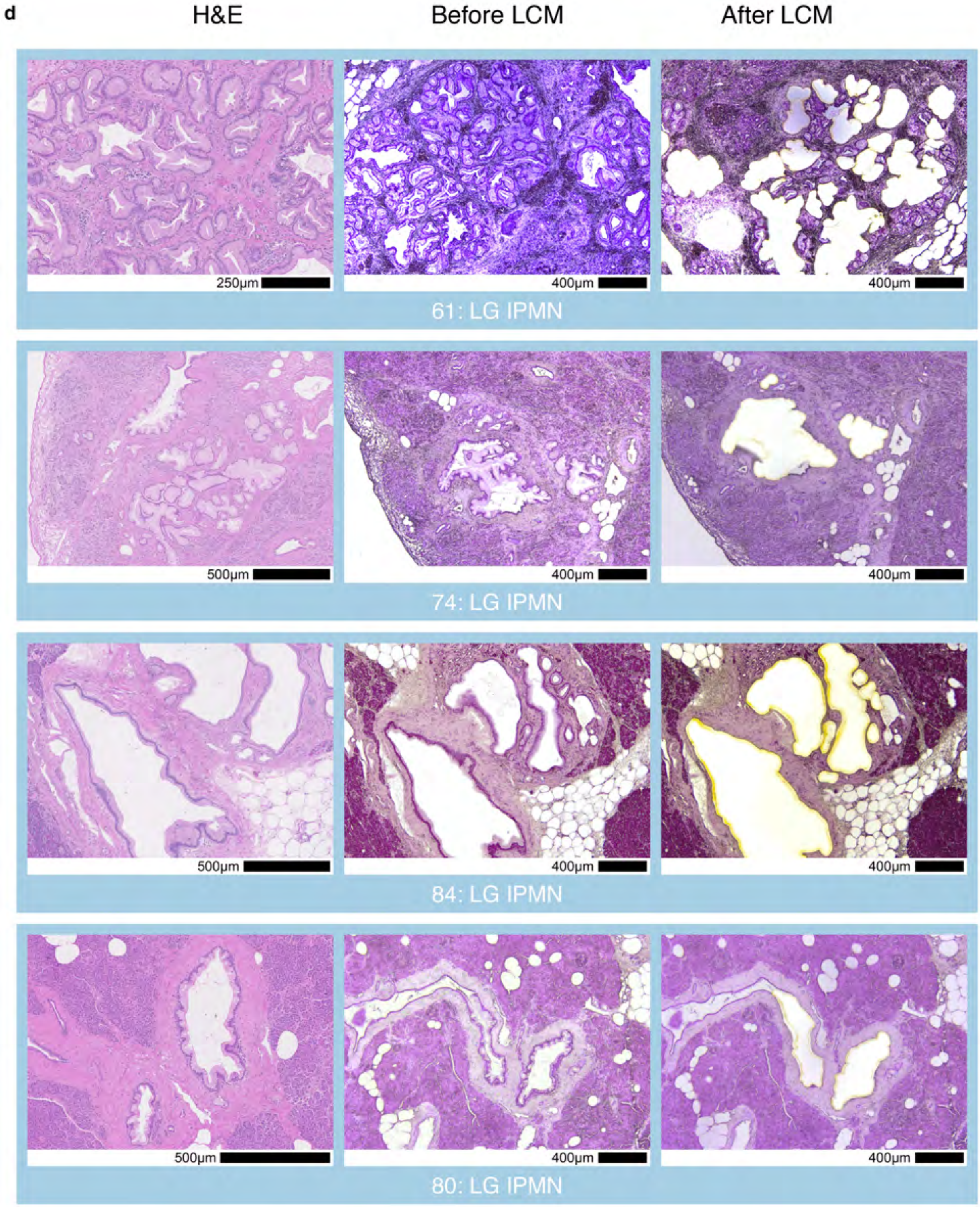

Supplementary Figure 14. Somatic mutations, phylogeny and laser capture microdissection in MTP19. 14d. Representative images of neoplastic tissue stained by hematoxylin and eosin (H&E), as well as isolated regions before and after laser capture microdissection are shown.

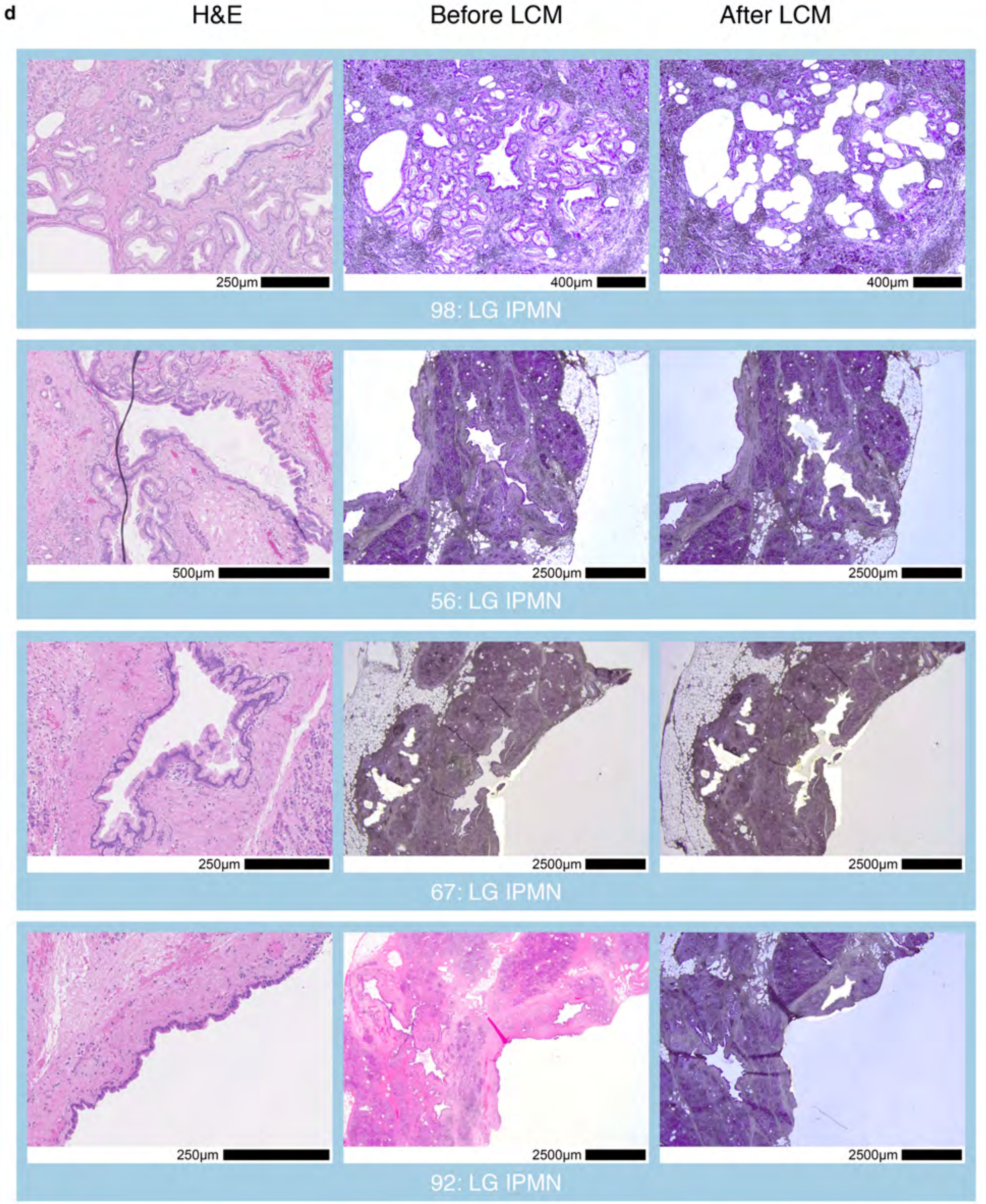

Supplementary Figure 14. Somatic mutations, phylogeny and laser capture microdissection in MTP19. 14d. Representative images of neoplastic tissue stained by hematoxylin and eosin (H&E), as well as isolated regions before and after laser capture microdissection are shown.

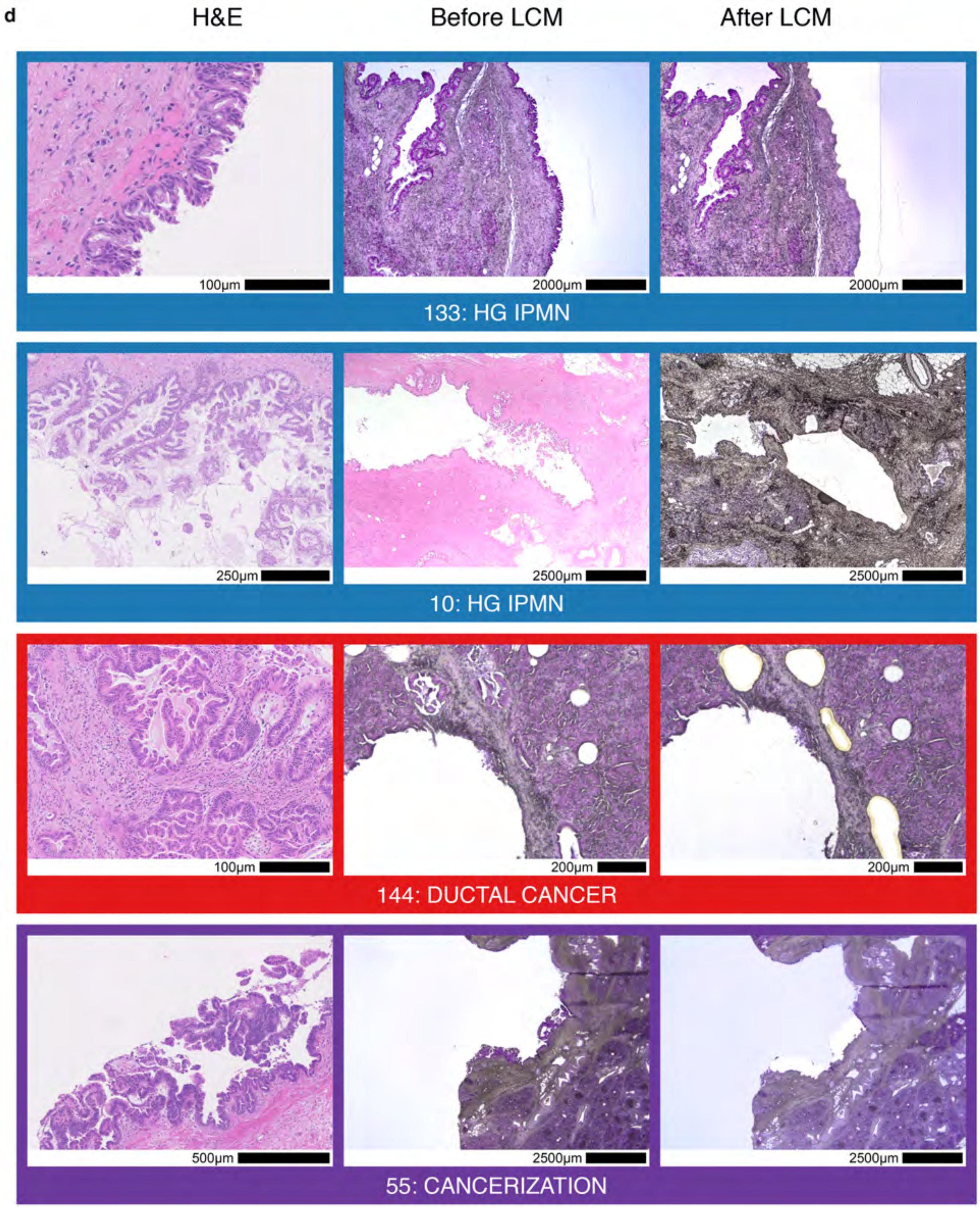

Supplementary Figure 14. Somatic mutations, phylogeny and laser capture microdissection in MTP19. 14d. Representative images of neoplastic tissue stained by hematoxylin and eosin (H&E), as well as isolated regions before and after laser capture microdissection are shown.

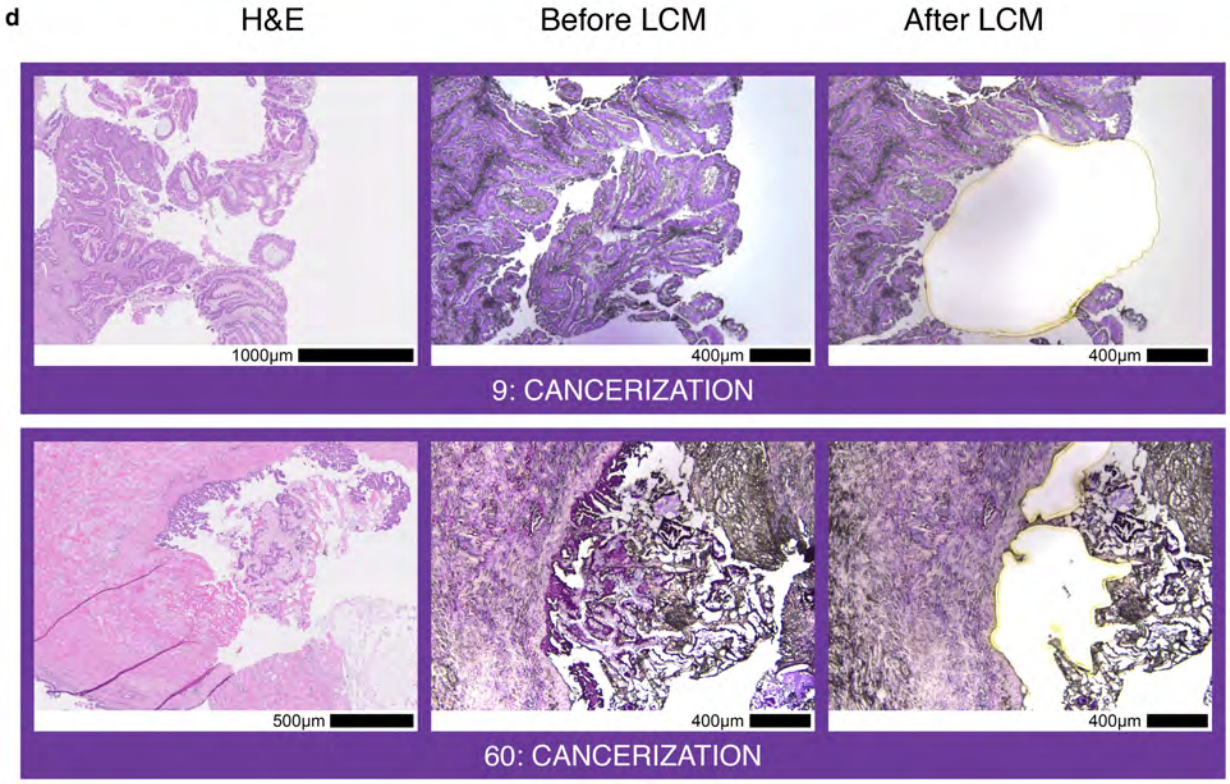

Supplementary Figure 15. Somatic mutations and phylogeny in MTP23. 15a. Mutations (rows) identified in the different samples (columns) in MTP23. Sample and mutation characteristics are described by the legend. The type of sequencing analysis (targeted or whole exome sequencing) performed for each sample is indicated in a track on the bottom. 15b. The inferred tumor phylogeny. The pathological characteristics for the clones are indicated by the color of the line and driver mutations are indicated at branch points. 15c. Comparison of variant allele frequencies (VAFs) in precancer/cancer sample pair. Sample and mutation types are indicated by the colors in the legend. High VAFs of mutations shared in IPMN/MCN and cancer samples demonstrate clonal relatedness and exclude the possibility of contamination by minute amounts of cells.

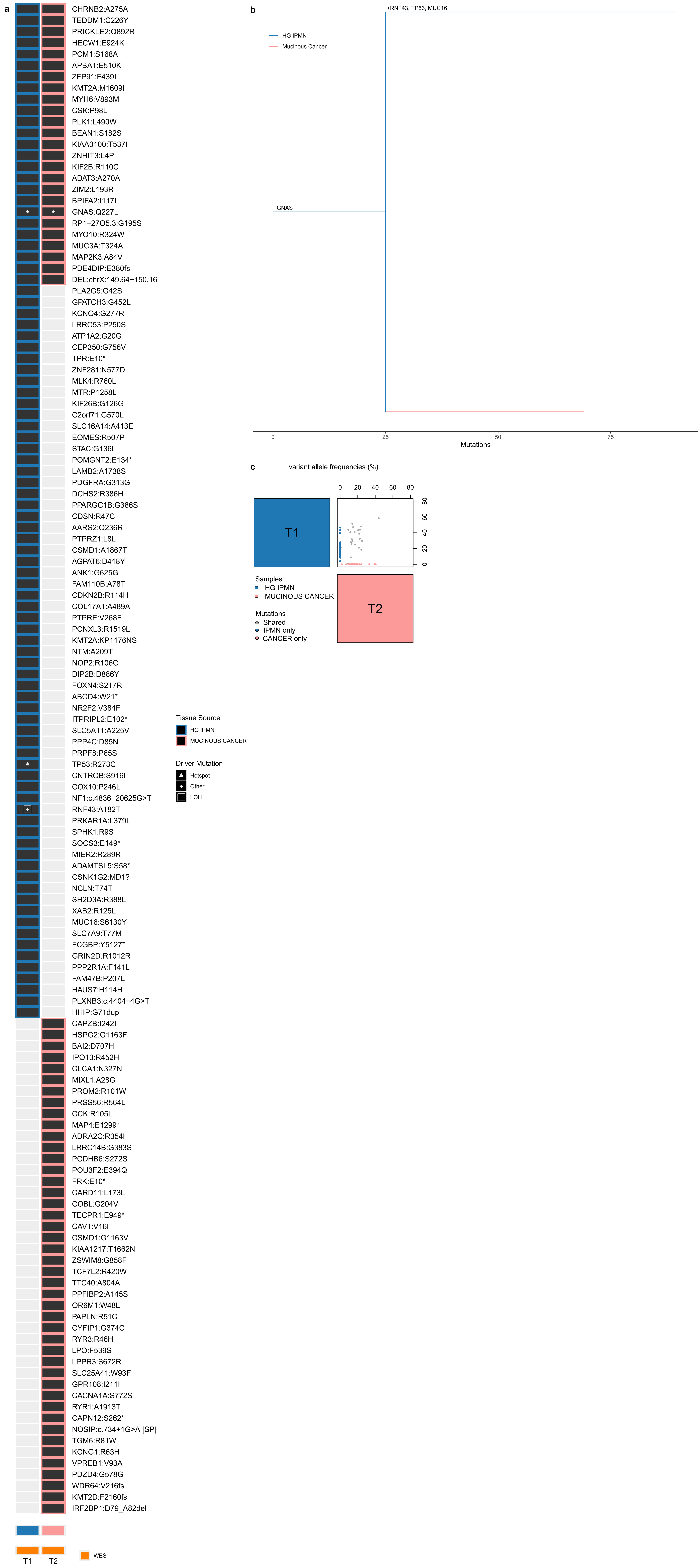

Supplementary Figure 16. Somatic mutations, phylogeny and laser capture microdissection in MTP24. 16a. Mutations (rows) identified in the different samples (columns) in MTP24. Sample and mutation characteristics are described by the legend. The type of sequencing analysis (targeted or whole exome sequencing) performed for each sample is indicated in a track on the bottom. 16b. The inferred tumor phylogeny. The pathological characteristics for the clones are indicated by the color of the line and driver mutations are indicated at branch points. 16c. Comparison of variant allele frequencies (VAFs) in sample pairs. Each plot shows the VAFs of the corresponding samples indicated on the horizontal and vertical axes. Sample and mutation types are indicated by the colors in the legend. High VAFs of mutations shared in IPMN/MCN and cancer samples demonstrate clonal relatedness and exclude the possibility of contamination by minute amounts of cells.

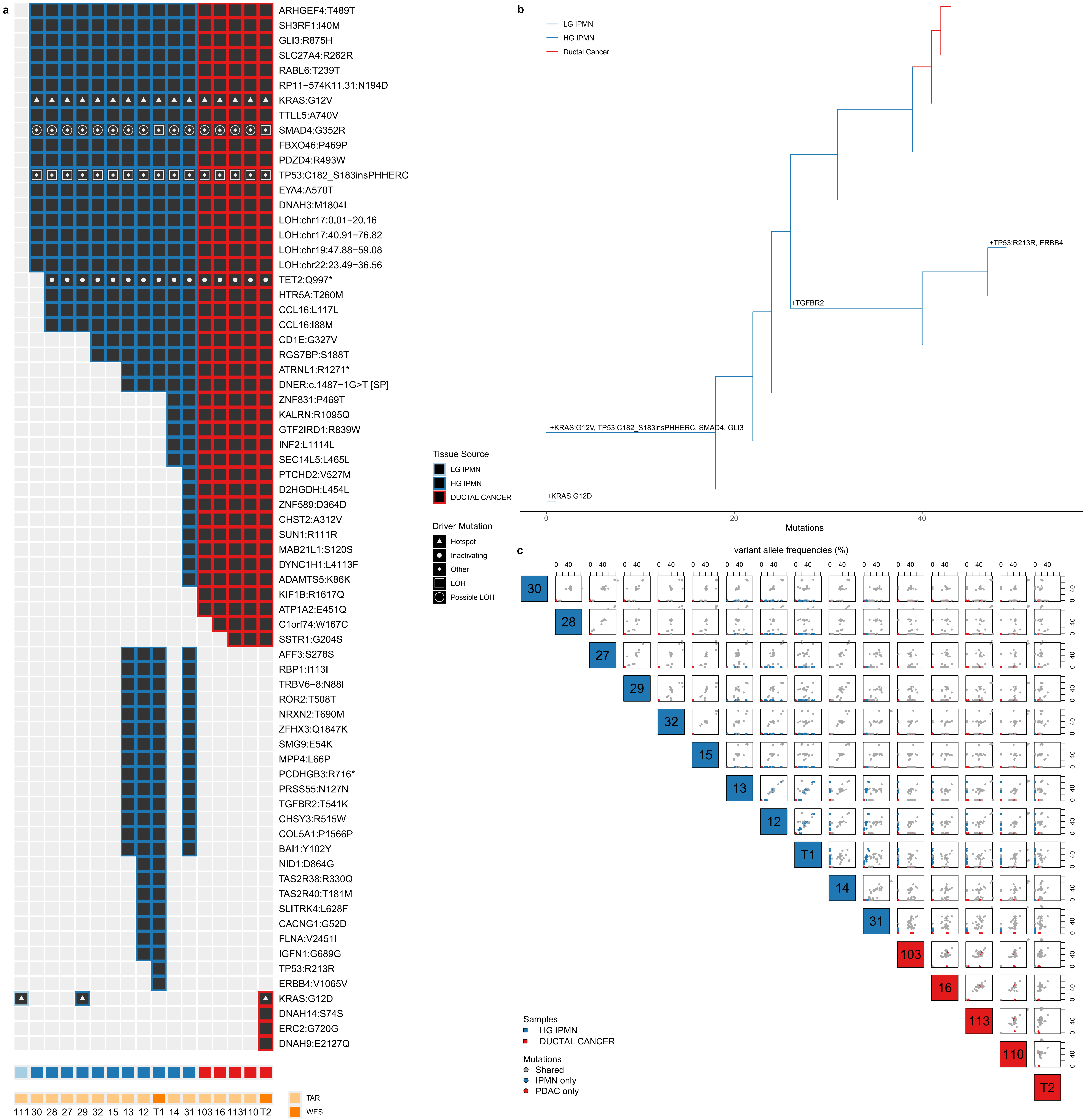

Supplementary Figure 16. Somatic mutations, phylogeny and laser capture microdissection in MTP24. 16d. Representative images of neoplastic tissue stained by hematoxylin and eosin (H&E), as well as isolated regions before and after laser capture microdissection are shown.

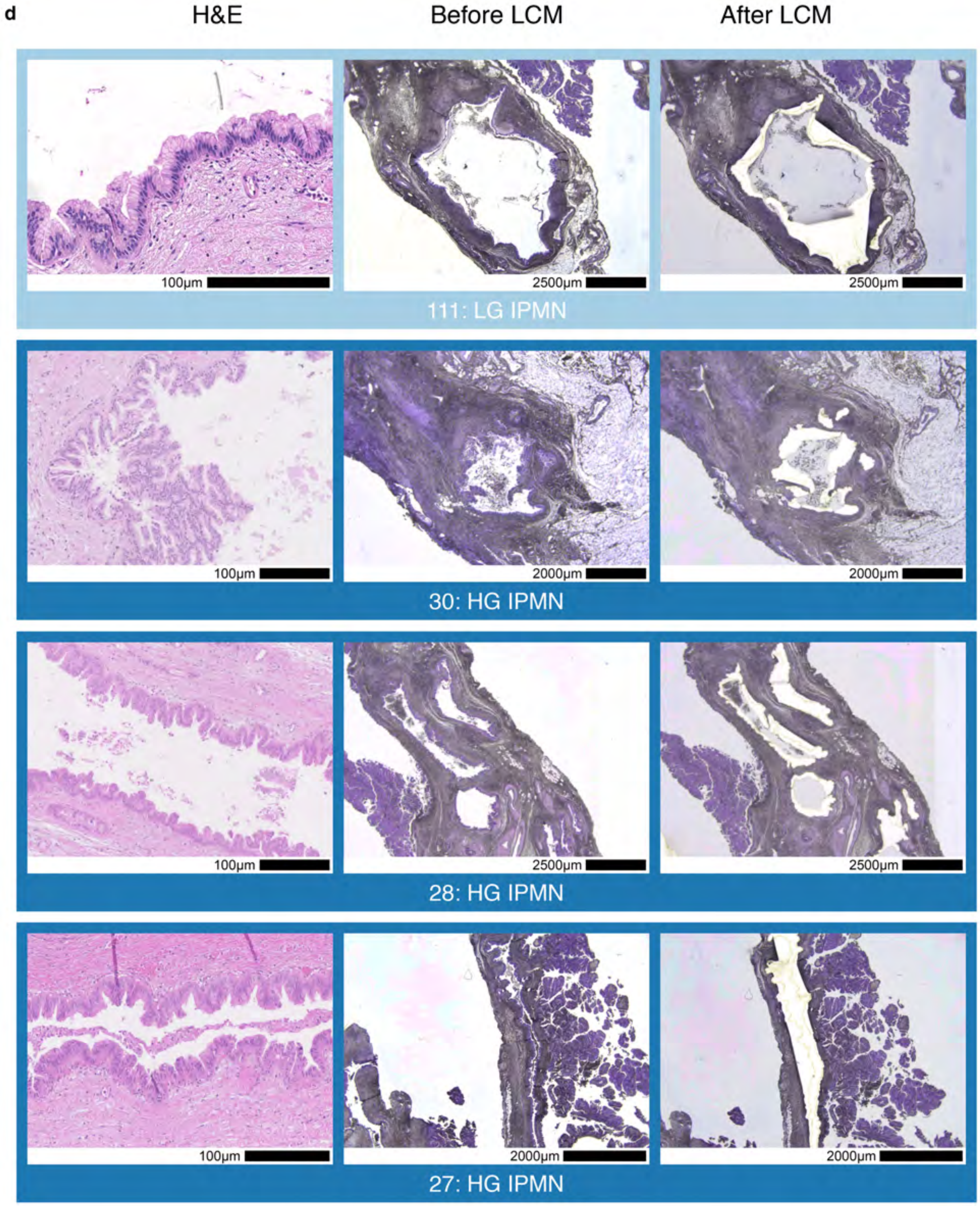

Supplementary Figure 16. Somatic mutations, phylogeny and laser capture microdissection in MTP24. 16d. Representative images of neoplastic tissue stained by hematoxylin and eosin (H&E), as well as isolated regions before and after laser capture microdissection are shown.

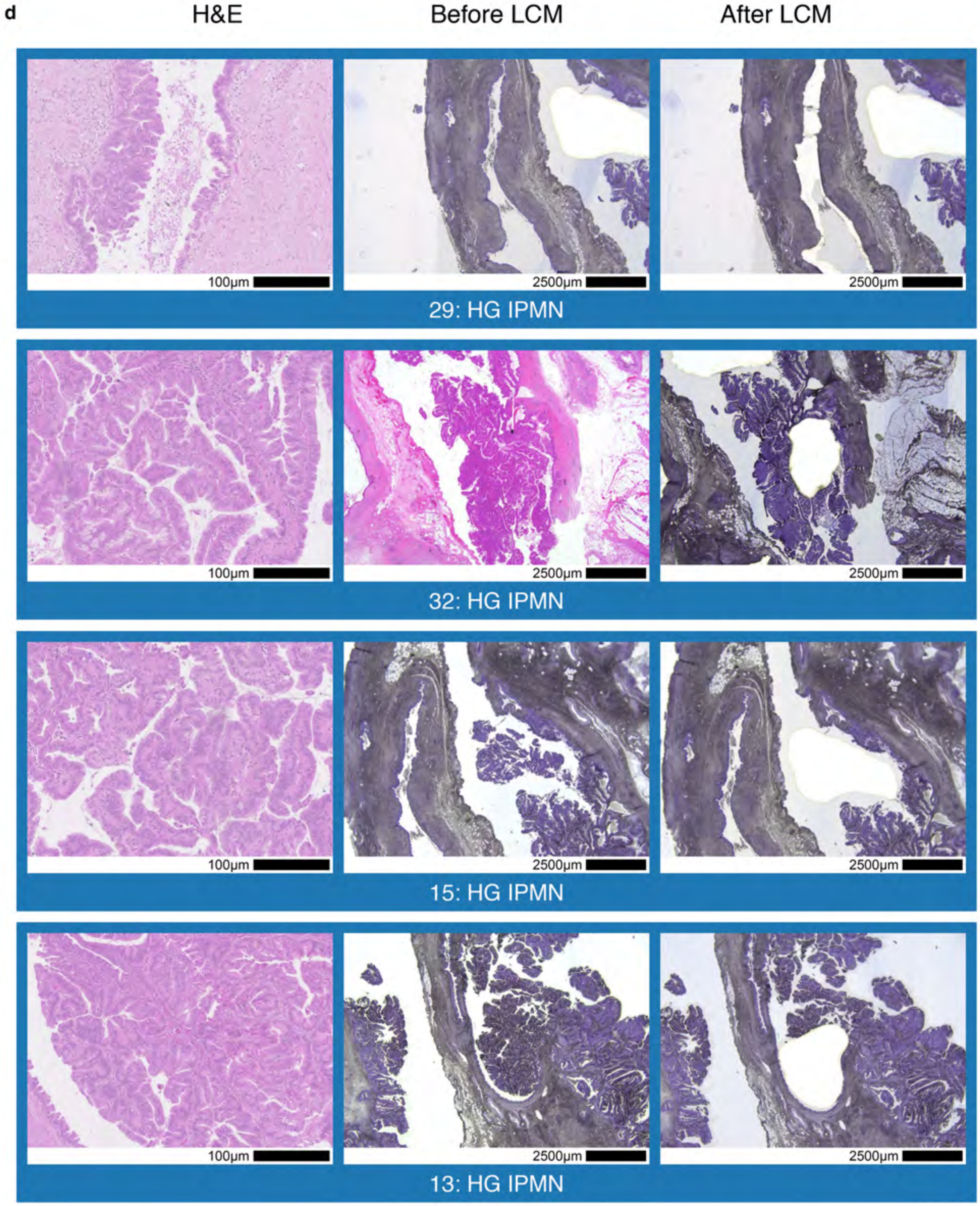

Supplementary Figure 16. Somatic mutations, phylogeny and laser capture microdissection in MTP24. 16d. Representative images of neoplastic tissue stained by hematoxylin and eosin (H&E), as well as isolated regions before and after laser capture microdissection are shown.

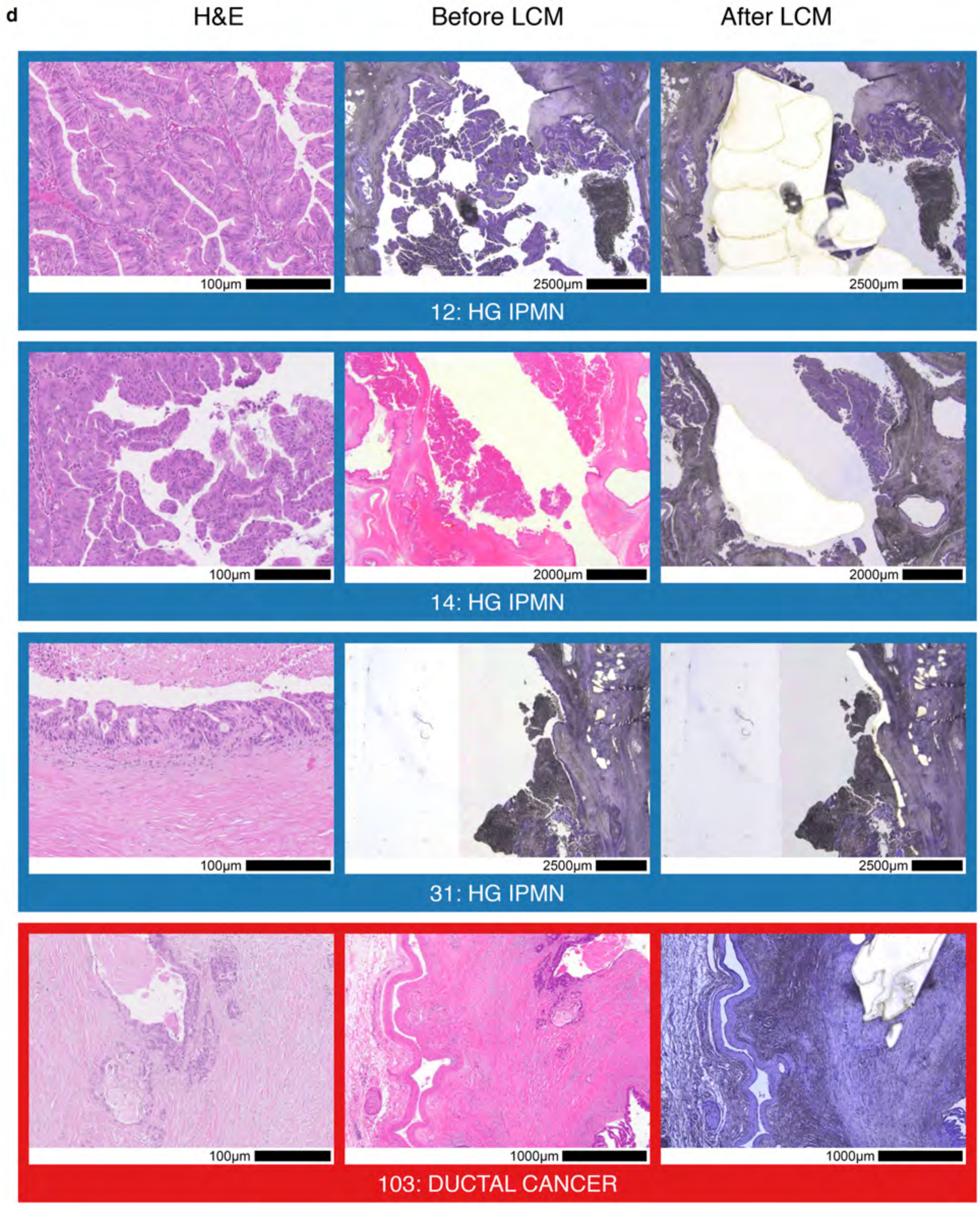

Supplementary Figure 16. Somatic mutations, phylogeny and laser capture microdissection in MTP24. 16d. Representative images of neoplastic tissue stained by hematoxylin and eosin (H&E), as well as isolated regions before and after laser capture microdissection are shown.

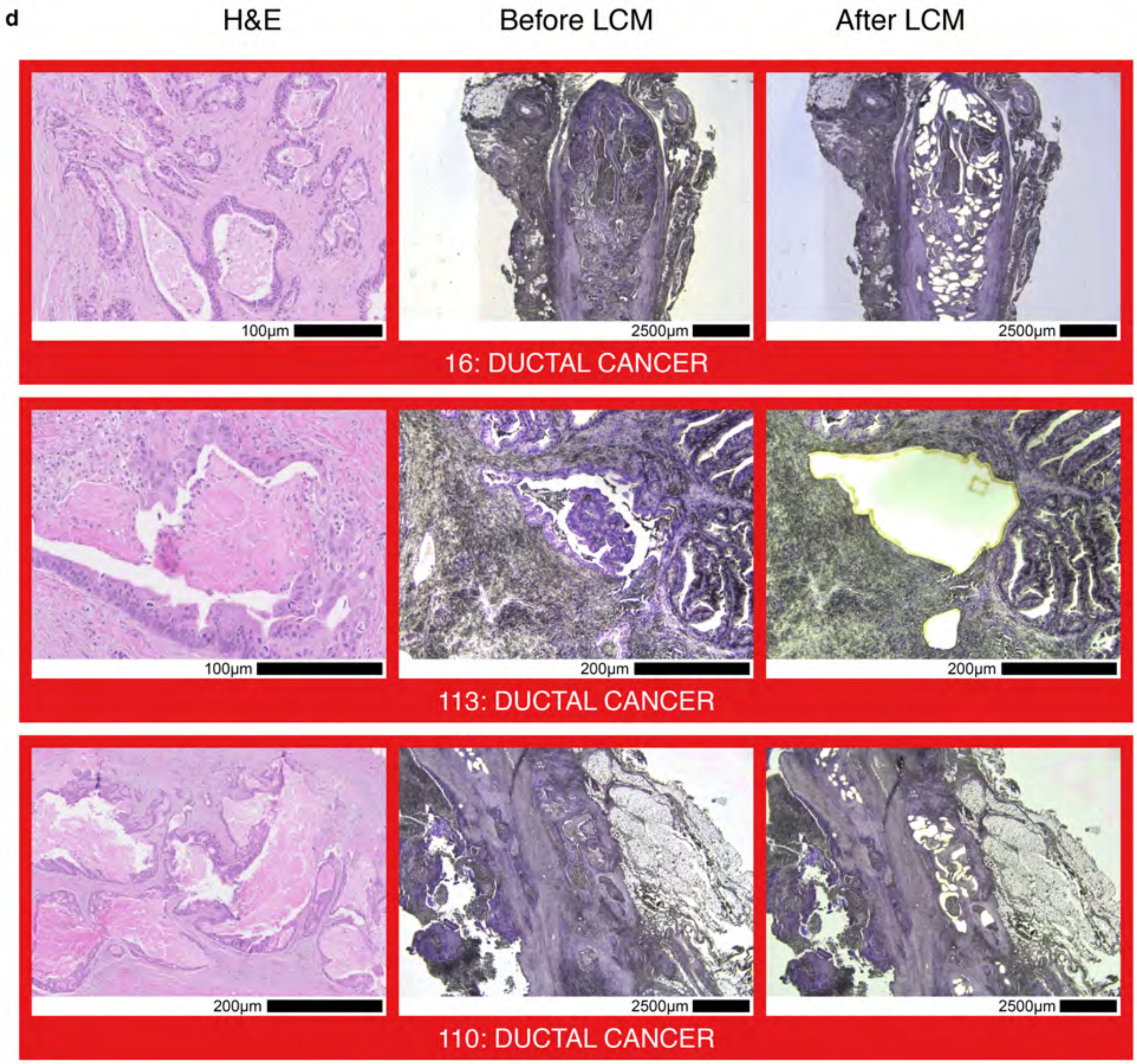

Supplementary Figure 17. Somatic mutations and phylogeny in MTP26. 17a. Mutations (rows) identified in the different samples (columns) in MTP26. Sample and mutation characteristics are described by the legend. The type of sequencing analysis (targeted or whole exome sequencing) performed for each sample is indicated in a track on the bottom. 17b. The inferred tumor phylogeny. The pathological characteristics for the clones are indicated by the color of the line and driver mutations are indicated at branch points. 17c. Comparison of variant allele frequencies (VAFs) in precancer/cancer sample pair. Sample and mutation types are indicated by the colors in the legend. High VAFs of mutations shared in IPMN/MCN and cancer samples demonstrate clonal relatedness and exclude the possibility of contamination by minute amounts of cells.

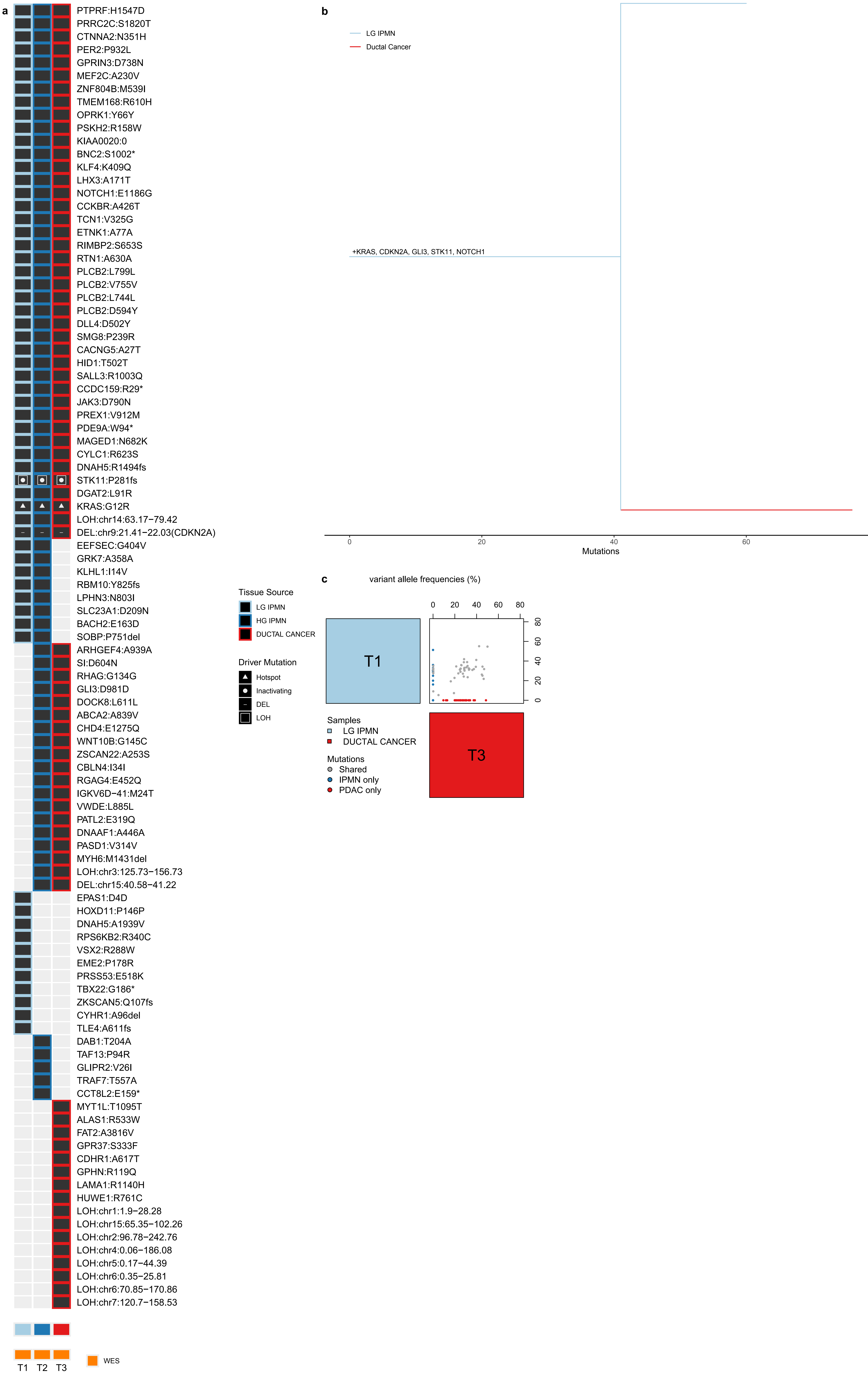

Supplementary Figure 18. Somatic mutations and phylogeny in MTP30. 18a. Mutations (rows) identified in the different samples (columns) in MTP30. Sample and mutation characteristics are described by the legend. The type of sequencing analysis (targeted or whole exome sequencing) performed for each sample is indicated in a track on the bottom. 18b. The inferred tumor phylogeny. The pathological characteristics for the clones are indicated by the color of the line and driver mutations are indicated at branch points. 18c. Comparison of variant allele frequencies (VAFs) in precancer/cancer sample pair. Sample and mutation types are indicated by the colors in the legend. High VAFs of mutations shared in IPMN/MCN and cancer samples demonstrate clonal relatedness and exclude the possibility of contamination by minute amounts of cells.

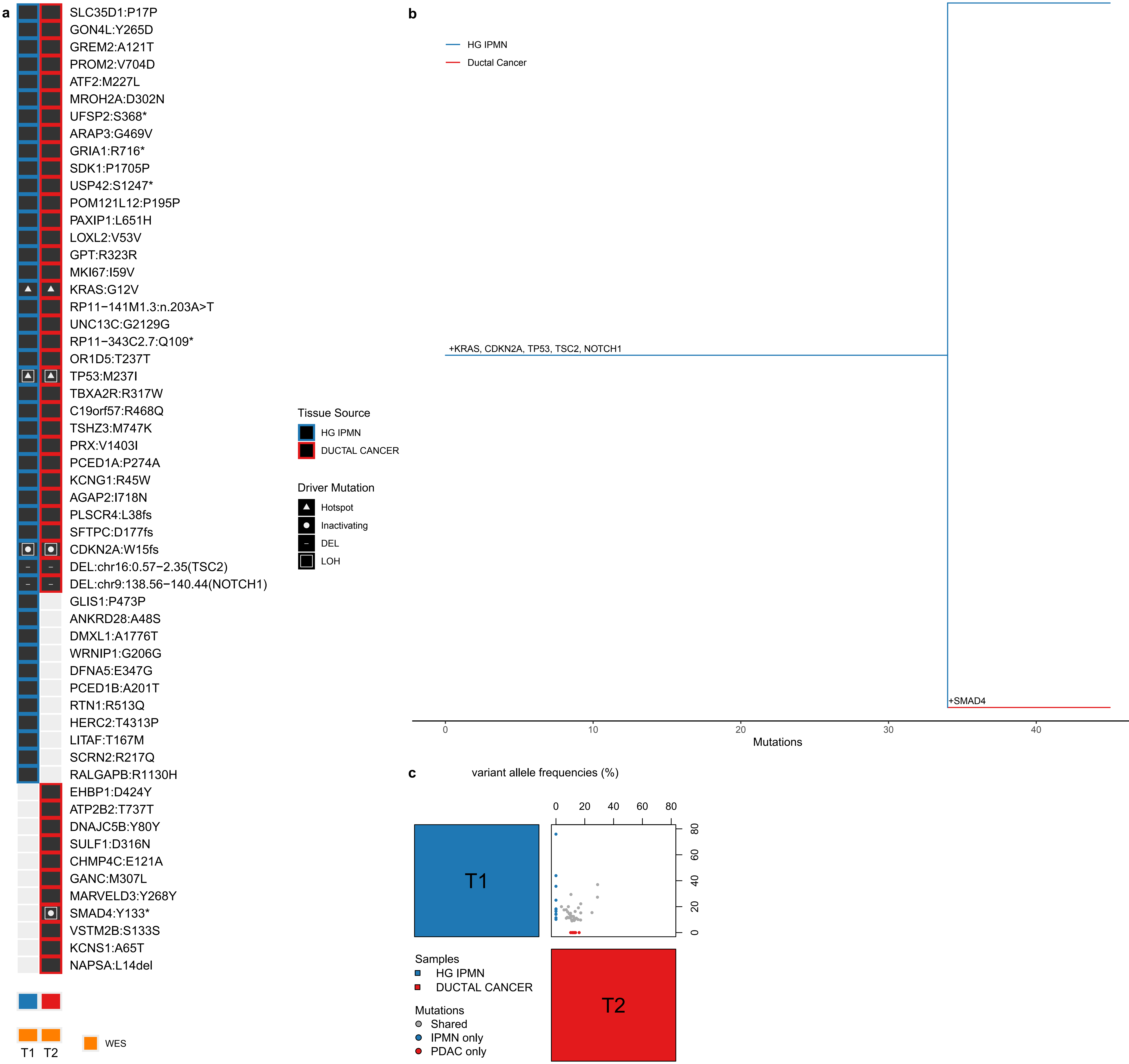

Supplementary Figure 19. Analysis of mutational signatures in whole exome sequencing data. Per case (columns), the color gradient shows the fractional contribution of the different mutational signatures (rows) to the total amount of single nucleotide variants.

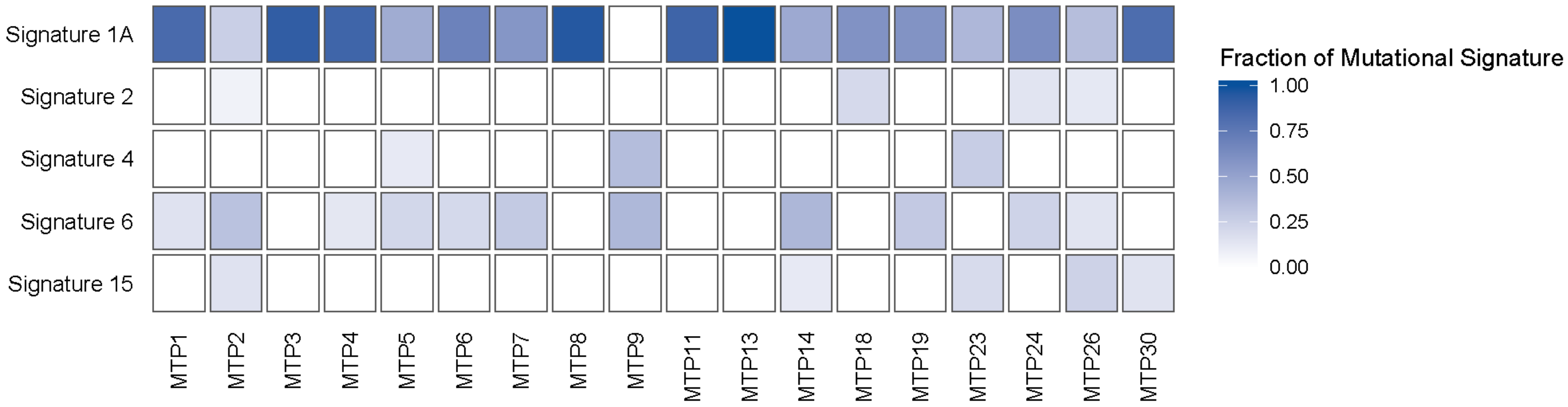

Supplementary Figure 20. Analysis of timing of malignant progression. We modeled the number of additional mutations acquired in a PDAC for 17 patients by fitting ~900 Bayesian hierarchical models, each predicated on a different unobserved mutation rate (y-axis) that was assumed to be constant during the IPMN to PDAC transition. The shaded band depicts the 90% posterior credible interval for the number of years (x-axis) to the development of the PDAC. The error bar for each patient indicates the 0.05 and 0.95 quantiles of the distribution of posterior medians from the different Bayesian models.

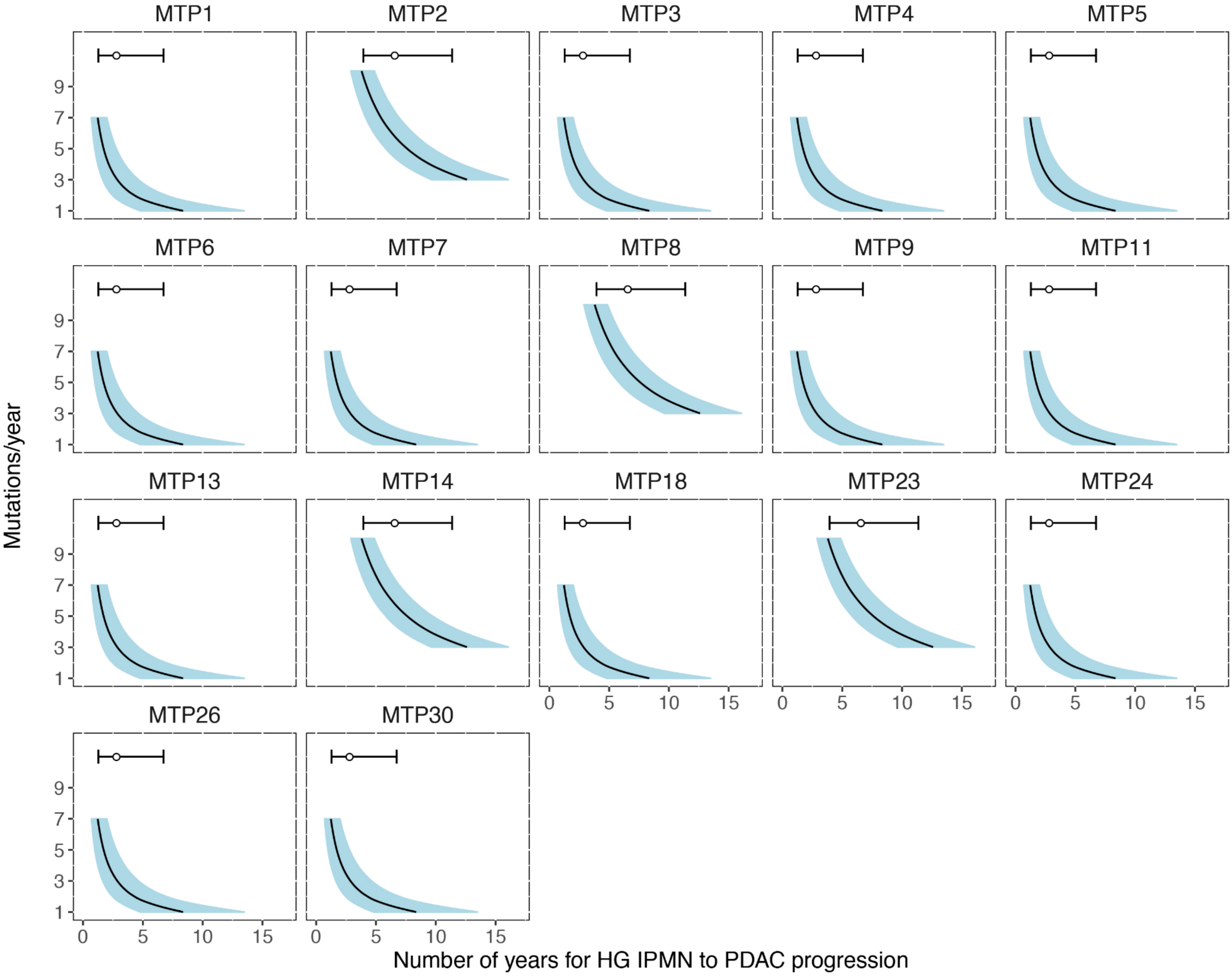

Supplement: Supplementary file 1 — Supplementary Information [file 41467_2020_17917_MOESM1_ESM.pdf]
