# Supplementary material for: Genomic characterization of malignant progression in neoplastic pancreatic cysts
Source: Nat Commun. 2020 Aug 14;11:4085. doi: 10.1038/s41467-020-17917-8 (PMC7428044; doi:10.1038/s41467-020-17917-8)
Supplement: Supplementary file 3 — Description of Additional Supplementary Files [file 41467_2020_17917_MOESM3_ESM.docx]

**Description of Additional Supplementary Material**

**Filename: Supplementary Data 1**

Description: Summary of patient and sample characteristics. Clinical and pathological characteristics of the IPMN tissue samples analyzed by targeted and whole exome sequencing.

**Filename: Supplementary Data 2**

Description: Summary of next generation sequencing analyses. Data summary of technical and quality metrics of next generation sequencing experiments.

**Filename: Supplementary Data 3**

Description: Genomic regions analyzed in targeted sequencing panel. Genomic positions of regions captured in targeted next generation sequencing panel.

**Filename: Supplementary Data 4**

Description: Somatic mutations identified in targeted and whole exome sequencing. Gene names, genomic positions, sequence alterations, and mutation types for all somatic mutations identified in whole exome and targeted sequencing.

**Filename: Supplementary Data 5**

Description: Regions of focal copy number alteration identified in whole exome sequencing. Genomic positions, fold change, and included genes in focal somatic copy number alterations in whole exome sequencing data.

**Filename: Supplementary Data 6**

Description: Regions of loss of heterozygosity identified in targeted and whole exome sequencing. Genomic positions and allele frequencies of regions of loss of heterozygosity identified in both targeted and whole exome sequencing analyses.

**Filename: Supplementary Data 7**

Description: *SMAD4* alterations identified in targeted and whole exome sequencing. Summary of *SMAD4* alterations identified in all analyzed samples.
