# Supplementary material for: Genomic characterization of malignant progression in neoplastic pancreatic cysts
Source: Nat Commun. 2020 Aug 14;11:4085. doi: 10.1038/s41467-020-17917-8 (PMC7428044; doi:10.1038/s41467-020-17917-8)
Supplement: Supplementary file 11 — Reporting Summary [file 41467_2020_17917_MOESM11_ESM.pdf]

nature research

Corresponding author(s): Dr. Laura D. Wood

Last updated by author(s): Jun 29, 2020

Reporting Summary

Nature Research wishes to improve the reproducibility of the work that we publish. This form provides structure for consistency and transparency in reporting. For further information on Nature Research policies, see our Editorial Policies and the Editorial Policy Checklist.

Statistics

For all statistical analyses, confirm that the following items are present in the figure legend, table legend, main text, or Methods section.

n/a	Confirmed	
☐	☒	The exact sample size (*n*) for each experimental group/condition, given as a discrete number and unit of measurement
☐	☒	A statement on whether measurements were taken from distinct samples or whether the same sample was measured repeatedly
☒	☐	The statistical test(s) used AND whether they are one- or two-sided<br>*Only common tests should be described solely by name; describe more complex techniques in the Methods section.*
☒	☐	A description of all covariates tested
☒	☐	A description of any assumptions or corrections, such as tests of normality and adjustment for multiple comparisons
☒	☐	A full description of the statistical parameters including central tendency (e.g. means) or other basic estimates (e.g. regression coefficient) AND variation (e.g. standard deviation) or associated estimates of uncertainty (e.g. confidence intervals)
☒	☐	For null hypothesis testing, the test statistic (e.g. *F*, *t*, *r*) with confidence intervals, effect sizes, degrees of freedom and *P* value noted<br>*Give P values as exact values whenever suitable.*
☐	☒	For Bayesian analysis, information on the choice of priors and Markov chain Monte Carlo settings
☒	☐	For hierarchical and complex designs, identification of the appropriate level for tests and full reporting of outcomes
☒	☐	Estimates of effect sizes (e.g. Cohen's *d*, Pearson's *r*), indicating how they were calculated

Our web collection on statistics for biologists contains articles on many of the points above.

Software and code

Policy information about availability of computer code

Data collection	No software was used to collect data.
Data analysis	Analysis of the sequencing data was done with: CASAVA (v1.8), BWA (v0.7.17-r1188), picard (v2.18.1), GATK (v3.7) (which also contains Mutect2), strelka (v2.9.9), manta (v1.4.0), BLAT (v36), deconstructSigs (v1.8.0), FACETS (v0.5.14)<br>Phylogenetic analysis: SCHISM (v1.1.2), PHYLIP (v3.695), ggtree (v1.4.11)<br>Timing of malignant progression was done with: JAGS v4.3.0 (R package). The custom code for this analysis is available at https://gitlab.com/cancer-genomx/ipmn-timing

For manuscripts utilizing custom algorithms or software that are central to the research but not yet described in published literature, software must be made available to editors and reviewers. We strongly encourage code deposition in a community repository (e.g. GitHub). See the Nature Research guidelines for submitting code & software for further information.

Data

Policy information about availability of data

All manuscripts must include a data availability statement. This statement should provide the following information, where applicable:
- Accession codes, unique identifiers, or web links for publicly available datasets
- A list of figures that have associated raw data
- A description of any restrictions on data availability

When permitted by the relevant IRBs (77 samples), whole exome and targeted sequencing data has been deposited in the European Genome-phenome Archive with accession EGAS00001004473.

Field-specific reporting

Please select the one below that is the best fit for your research. If you are not sure, read the appropriate sections before making your selection.

- [x] Life sciences
- [ ] Behavioural & social sciences
- [ ] Ecological, evolutionary & environmental sciences

For a reference copy of the document with all sections, see nature.com/documents/nr-reporting-summary-flat.pdf

Life sciences study design

All studies must disclose on these points even when the disclosure is negative.

Sample size	Pancreatic neoplastic cysts with small tumors are very rare. We could only find 18 cases in the pathology archives of our hospital and collaborators. No power-analyses were done.
Data exclusions	We did not allow tumors to be large, as increasing volume of the tumors could result in the further accumulation of mutations, which could make it more difficult to find the molecular event that resulted in invasion of the neoplastic cyst.
Replication	Each neoplastic sample was sequenced once. Normal samples were sequenced twice: once with the whole exome enrichment panel and once with the targeted enrichment panel.
Randomization	This is not relevant to the study: this study is a cross-sectional, case-only study of cancers in patients with pancreatic cancers. All samples and all patients were treated the same way.
Blinding	This is not relevant to the study: the methods in this study have been established and do not rely on human interpretation. All samples were treated in the same way and the protocol has been described in the methods section. The study reports the results from unbiased bioinformatic and statistical analyses.

Reporting for specific materials, systems and methods

We require information from authors about some types of materials, experimental systems and methods used in many studies. Here, indicate whether each material, system or method listed is relevant to your study. If you are not sure if a list item applies to your research, read the appropriate section before selecting a response.

Materials & experimental systems

n/a	Involved in the study	
x		Antibodies
x		Eukaryotic cell lines
x		Palaeontology and archaeology
x		Animals and other organisms
	x	Human research participants
x		Clinical data
x		Dual use research of concern

Methods

n/a	Involved in the study	
x		ChIP-seq
x		Flow cytometry
x		MRI-based neuroimaging

Human research participants

Policy information about studies involving human research participants

Population characteristics	Most patients were older, as patients who are older are at higher risk to develop pancreatic cancer. Both males and females were included.
Recruitment	Pathology archives of multiple institutions were searched for cases that matched our inclusion criteria. Pathologic assessment was done by multiple experienced pathologists with extensive expertise in GI pathology. Samples with large tumors were excluded for reasons explained earlier. This method of patient-recruitment has been used with many other sequencing studies. We do not expect a relationship between patients included in the study and the intended study-population. Our results are similar to other studies that sequenced the same lesions. We do not expect any other bias that could affect the results described.
Ethics oversight	An IRB protocol was submitted and approved at the Johns Hopkins University. Additional IRB protocols were submitted and approved in collaborating institutions when a local IRB was required. Samples came from the Johns Hopkins Hospital, Baltimore (United States); University Medical Center, Utrecht (The Netherlands); University and Hospital Trust, Verona (Italy); Asan Medical Center, Seoul (Republic of Korea); National Cancer Center Hospital, Tokyo (Japan); Royal North Shore Hospital, Sydney (Australia), University Hospital, Ghent (Belgium); Academic Medical Center, Amsterdam (The Netherlands); Laboratory for Pathology Eastern Netherlands, Hengelo (The Netherlands); Thomas Jefferson University, Philadelphia (United States); Aichi Cancer Center Hospital, Nagoya (Japan); Medica Sur Clinic and Foundation, Mexico City (Mexico); Emory University Hospital, Altanta (United States)

Note that full information on the approval of the study protocol must also be provided in the manuscript.
